# Effect of sodium-glucose cotransporter-2 inhibitors on fracture risk in patients with type 1 diabetes receiving insulin-based therapy: a meta-analysis

**DOI:** 10.7717/peerj.21087

**Published:** 2026-04-16

**Authors:** Huimei Chen, Zhe Lin, Ziyi Liu, Shaoming Li, Peng Xue, Kangxu Wei, Menghan Zhang, Jiarui Cui, Wenxuan Liu, Na Wang

**Affiliations:** 1 Hebei Medical University, Shijiazhuang, China; 2 Department of Endocrinology, Hebei Medical University Third Hospital, Shijiazhuang, China; 3 Department of Orthopedic Surgery, The Hebei Medical University Third Hospital, Shijiazhuang, China; 4 Department of Endocrinology and Nephrology, Shenzhou Hospital, Shenzhou, China; 5 Department of Epidemiology and Statistics, School of Public Health, Hebei Medical University, Hebei Key Laboratory of Environment and Human Health, Shijiazhuang, China

**Keywords:** SGLT2i, T1DM, Fracture, Meta-analysis

## Abstract

**Background:**

Sodium-glucose cotransporter-2 inhibitors (SGLT2i), a novel class of antihyperglycemic agents, have raised concerns regarding bone safety. This meta-analysis aimed to evaluate the specific effect of adjunctive SGLT2i therapy on fracture risk in patients with type 1 diabetes mellitus (T1DM).

**Methods:**

We systematically searched four databases (PubMed, Embase, Cochrane Library and Web of Science Core Collection) to identifyall eligible randomized controlled trials (RCTs) investigating SGLT2i as adjunctive therapy to insulin in T1DM. Fracture risk was defined as primary outcome, while glycemic parameters, non-glycemic outcomes, and other safety index serving as secondary endpoints. Pooled ORs (95% CIs) were calculated, with dose-stratified subgroup analyses. Risk of bias was assessed using the Cochrane Collaboration Risk-of-Bias tool (RoB 2).

**Results:**

Our analysis included 10 RCTs comprising 6,731 T1DM patients. All included studies were deemed to be at low or moderate risk of bias. Pooled analysis revealed no significant association between SGLT2i use and fracture risk (OR 0.98, 95% CI [0.63–1.51]). This null finding remained consistent across subgroup analyses. Fracture odds ratios in the low-, moderate-, and high-dose subgroups were 0.78 (95% CI [0.11–5.58]), 1.08 (95% CI [0.55–2.11]), and 0.90 (95% CI [0.50–1.63]), respectively. SGLT2i significantly improved glycemic control, including HbA1c, fasting plasma glucose, and time in range. It also reduced body weight and blood pressure. However, SGLT2i treatment increased the risk of diabetic ketoacidosis (OR 3.52, 95% CI [2.16–5.71]) and genital tract infections (OR 3.69, 95% CI [2.85–4.78]).

**Conclusion:**

This meta-analysis provides reassuring evidence that adjunctive SGLT2 inhibitor use is not associated with increased fracture risk in insulin-treated patients with T1DM patients. Nonetheless, the substantially elevated risks of diabetic ketoacidosis and genital tract infections necessitate vigilant clinical monitoring and risk mitigation strategies to ensure safe use of these agents.

## Introduction

The global incidence and prevalence of type 1 diabetes mellitus (T1DM) have been rising, with the overall annual incidence increasing by 2–3% (DiMeglio, Evans-Molina & Oram, 2018). T1DM was characterized by absolute insulin deficiency and resultant hyperglycemia, necessitating lifelong insulin replacement therapy. Despite continuous insulin supplementation, many patients continue to experience glycemic variability and remained at elevated risk of hypoglycemia (Janež et al., 2020). Originally developed for type 2 diabetes (T2DM), sodium-glucose cotransporter protein 2 inhibitor (SGLT2i) have recently been investigated as adjunctive therapy in T1DM to improve glycemic stability and reduce the insulin requirements. Mechanistically, SGLT2i decreased the renal threshold for glucose reabsorption, promoting urinary glucose excretion and reducing hyperglycemia independent of insulin action (Salvatore et al., 2022). Beyond their glucosuric effect, studies suggested that SGLT2i might confer additional metabolic benefits, including preservation of β-cell function and amelioration of insulin resistance (Kaneto et al., 2021). However, evidence indicated that SGLT2i might adversely influence bone metabolism, potentially through alterations in calcium-phosphate homeostasis and vitamin D metabolism (Youssef et al., 2023).

The role of SGLT2i in bone health in diabetic populations remained controversial. SGLT2i-induced glucosuria may disrupt renal calcium and phosphate handling (Jackson & Moseley, 2020). Ye et al. (2018) suggested that SGLT2i increased parathyroid hormone (PTH) and decreased 1,25-hydroxy vitamin D, both of which affected bone turnover accordingly. Indeed, a recent meta-analysis documented significant alterations in bone turnover markers among T2DM patients treated with SGLT2i (Wang et al., 2024). In contrast, a large randomized controlled trial (CREDENCE) found that canagliflozin, an SGLT2 inhibitor, exerted neutral effects of canagliflozin on fracture incidence in T2DM populations (Perkovic et al., 2019). Similarly, a comprehensive meta-analysis of 27 studies (20,895 T2DM patients) identified no significant association between SGLT2i and fracture risk (Li et al., 2019). Whether these findings extend to T1DM populations—who typically have longer disease duration, lower body mass index, and distinct pathophysiological profiles—remains uncertain.

In T1DM specifically, previous meta-analyses have predominantly focused on glycemic efficacy, hypoglycemia risk, and metabolic outcomes, with bone safety representing a critical evidence gap. Given the potential mechanistic concerns and the distinct demographic and clinical characteristics of T1DM patients, rigorous evaluation of fracture risk is warranted. This meta-analysis was conducted to quantify the major influence of SGLT2i on fracture risk in T1DM patients with insulin therapy to inform evidence-based clinical decision-making.

## Methods

We conducted this systematic review and meta-analysis in accordance with the Preferred Reporting Items for Systematic Reviews and Meta-Analyses (PRISMA) guidelines. The protocol was prospectively registered with PROSPERO (registration number: CRD420251001137).

### Data sources and search strategy

A pilot screening was conducted prior to protocol registration to validate the screening workflow (PROSPERO). No records were excluded at this stage. Following PROSPERO approval, the full documented screening process commenced. All final decisions were based on predefined criteria specified in the registered protocol to ensure transparency and minimize bias. From the inception of database to 24 October 2025, we systematically searched four databases (PubMed, Embase, Cochrane Library, Web of Science Core Collection). We used the following searching strategy that contained the terms of “sodium-glucose transporter 2, SGLT2, SGLT-2, Dapagliflozin, Canagliflozin, Empagliflozin, Sotagliflozin, and Ipragliflozin”. No language restrictions were imposed. Full searching strategies were detailed in Table S1.

### Study selection

Two investigators (LZY and LSM) independently assessed the eligibility of full-text articles according to the predefined criteria. If the two investigators had disagreements, senior researchers XP and LWX would resolve them by consensus. Inclusion criteria: (1) randomized controlled clinical trials (RCTs) evaluating the effects of SGLT2i on T1DM with insulin therapy; (2) reported outcomes including fracture incidence, bone turnover markers, or bone mineral density (BMD); (3) Participants aged ≥ 18 years; (4) intervention duration ≥ 12 weeks. Exclusion criteria: non-human studies; non-RCTs; interventions not involving SGLT2 inhibitors; non-T1DM patients; participants under 18 years old; conference abstract/editorials, reviews, case reports, commentaries, and articles that did not report outcomes or data of interests. For RCTs reported in multiple publications with varying follow-up durations, we included data from the longest follow-up period to avoid duplicate participant data (Musso et al., 2020).

### Outcomes

The primary outcome of interest was the risk of fracture. The secondary outcomes included glycemic efficacy: glycosylated hemoglobin type A1C (HbA1c), fasting plasma glucose (FPG), time-in-range (TIR, 70–180 mg/dl), and daily insulin requirements (total, basal, and bolus doses). The non-glycemic efficacy outcomes were as follows: bodyweight, systolic blood pressure (SysBP), diastolic blood pressure (DBP), estimated glomerular filtration rate (eGFR), urinary albumin to creatinine ratio (ACR), and diabetic eye disorders. Other safety outcomes were diabetic ketoacidosis (DKA), cardiovascular events (MACE), hypoglycemia, urinary tract infections (UTIs), and genital tract infections (GTIs) (Table 1).

**Table 1:** Glycemic and non-glycemic efficacy outcomes and safety outcomes evaluated in the meta-analysis.

**Glycemic efficacy outcomes**	
Outcome	Comments/description
HbA1c (%)	Changes in HbA1c (%) from baseline
Fasting plasma glucose (FPG)	Changes in FPG from baseline
Time-in-range (70–180 mg/dl) (%)	% of daily glucose reading between 70 and 180 mg/dL over each 24-h period during continuous glucose monitoring (CGM)
Hypoglycemia	Blood glucose <70 mg/dL
Daily total insulin dose (TID) changes	[(end-of treatment TID - initial TID)/initial TID] × 100%
Daily basal insulin dose (ID) changes	[(end-of treatment basal ID - initial basal ID)/initial basal ID] × 100%
Daily bolus ID changes	[(end-of treatment bolus ID - initial bolus ID)/initial bolus ID] × 100%
**Nonglycemic efficacy outcomes**	
Outcome	Comments/description
Body weight changes	[(end-of treatment body weight - initial body weight)/initial body weight] × 100%
SysBP changes (mmHg)	[(end-of treatment sysBP - initial sysBP)/initial sysBP] × 100%
Diastolic blood pressure (DBP) changes (mmHg)	[(end-of treatment dBP - initial dBP)/initial dBP] × 100%
eGFR changes(ml/min/1.73 m^2^)	[(end-of treatment eGFR - initial eGFR)/initial eGFR] × 100%
ACR changes(mg/g)	[(end-of treatment ACR - initial ACR)/initial ACR] × 100%
Diabetic eye disorders	Including development of hemorrhagic retinopathy/vitreous hemorrhage, retinal detachment, macular edema, glaucoma, or vision loss (as defined by the International Clinical Disease Severity Scale)
**Safety outcomes**	
Outcome	Comments/description
Bone fracture	Occurred after joining in the study
Definite diabetic ketoacidosis (DKA)	Anion-gap metabolic acidosis with ketone increases without a satisfactory alternative cause for anion-gap acidosis
Major Adverse Cardiovascular Events (MACE)	Cardiovascular death, myocardial infarction, stroke, hospitalization due to heart failure or unstable angina, or coronary revascularization
Urinary tract infections (UTIs)	Occurred after joining in the study
Genital tract infections (GTIs)	Occurred after joining in the study

### Extraction of data and assessment on risk of bias

Two investigators independently extracted detailed data using a predefined electronic data collection form according to the Cochrane Handbook for Systematic Reviews of Intervention. The qualities of RCTs were assessed across five domains (randomization process, deviations from the intended interventions, missing outcomes, measurement of the outcome, and selection of reported results), using the Cochrane Collaboration Risk-of-Bias tool (RoB2). Publication bias was evaluated by visually inspecting the symmetry of funnel plots.

Continuous variables were extracted and described with mean ± SD. Dichotomous variables were calculated with the number of occurrences. Four different SGLT2i (Sotagliflozin, Empagliflozin, Dapagliflozin, and Ipragliflozin) were involved in this study. These SGLT2i were sub-grouped based on the dosage in clinical trials as low, moderate, and high for analysis (low dose: Sotagliflozin 75 mg, Dapagliflozin 1 mg, Dapagliflozin 2.5 mg, Empagliflozin 2.5 mg, Ipragliflozin 25 mg; moderate dose: Sotagliflozin 200 mg, Canagliflozin 100 mg, Dapagliflozin 5 mg, Empagliflozin 10 mg, Ipragliflozin 50 mg; high dose: Sotagliflozin 400 mg, Canagliflozin 300 mg, Dapagliflozin 10 mg, Empagliflozin 25 mg, Ipragliflozin 100 mg). When trials were evaluated based on different SGLT2i dosages, we presented data separately for each dosage arm and split the sample size of the placebo group evenly among different dosage comparisons following the methodological guidelines outlined in sections 7.6 through 7.8 and 16.1.3 of the Cochrane Handbook for Systematic Reviews of Interventions.

### Data synthesis and analysis

We analyzed the data by Review Manager 5.4 software and reported according to the PRISMA guidelines. For dichotomous variables outcomes, the odds ratio (OR) with 95% confidence interval (CI) were pooled using the random-effects Mantel-Haenszel model. For continuous outcomes, mean average difference (MD) or standardized mean difference (SMD) with 95% CI were calculated by inverse variance random effects model. When continuous variables were reported in different units across studies, SMDs were used to allow for consistent pooling. A two-tailed *p* value ≤ 0.05 was considered statistically significant.

We conducted the Cochran’s Q test and assessed heterogeneity using I^2^, with *p* ≤ 0.1 was considered statistically significant in this part. The low, medium, and high levels of heterogeneity were determined by 25%, 50%, and 75%, respectively. When the results indicated a high statistical heterogeneity, we explored the underlying reasons, eliminated the source. Sensitivity analyses were performed by: (1) sequentially removing individual studies to identify influential outliers; (2) changing the effect size or the effect model to re-calculate the pooled estimates and explore sources heterogeneity. Analyses were recalculated after excluding studies identified as sources of heterogeneity.

### Certainty of evidence

The Grading of Recommendations Assessment, Development, and Evaluation (GRADE) was used for assessing the quality of evidence across outcomes in our study. We evaluated 13 outcomes as follows: HbA1c, FPG, TIR, hypoglycemia, daily total insulin dose, daily basal insulin dose, daily bolus insulin dose, body weight, sysBP, DBP, eGFR, ACR, and diabetic eye disorders; and five safety outcomes: bone fracture, DKA, MACE, UTIs, and GTIs. The evidence for each outcome was rated across the five GRADE domains: imprecision, inconsistency, risk of bias, indirectness, and reporting bias.

## Results

### Study selection and characteristics

**Figure 1:**
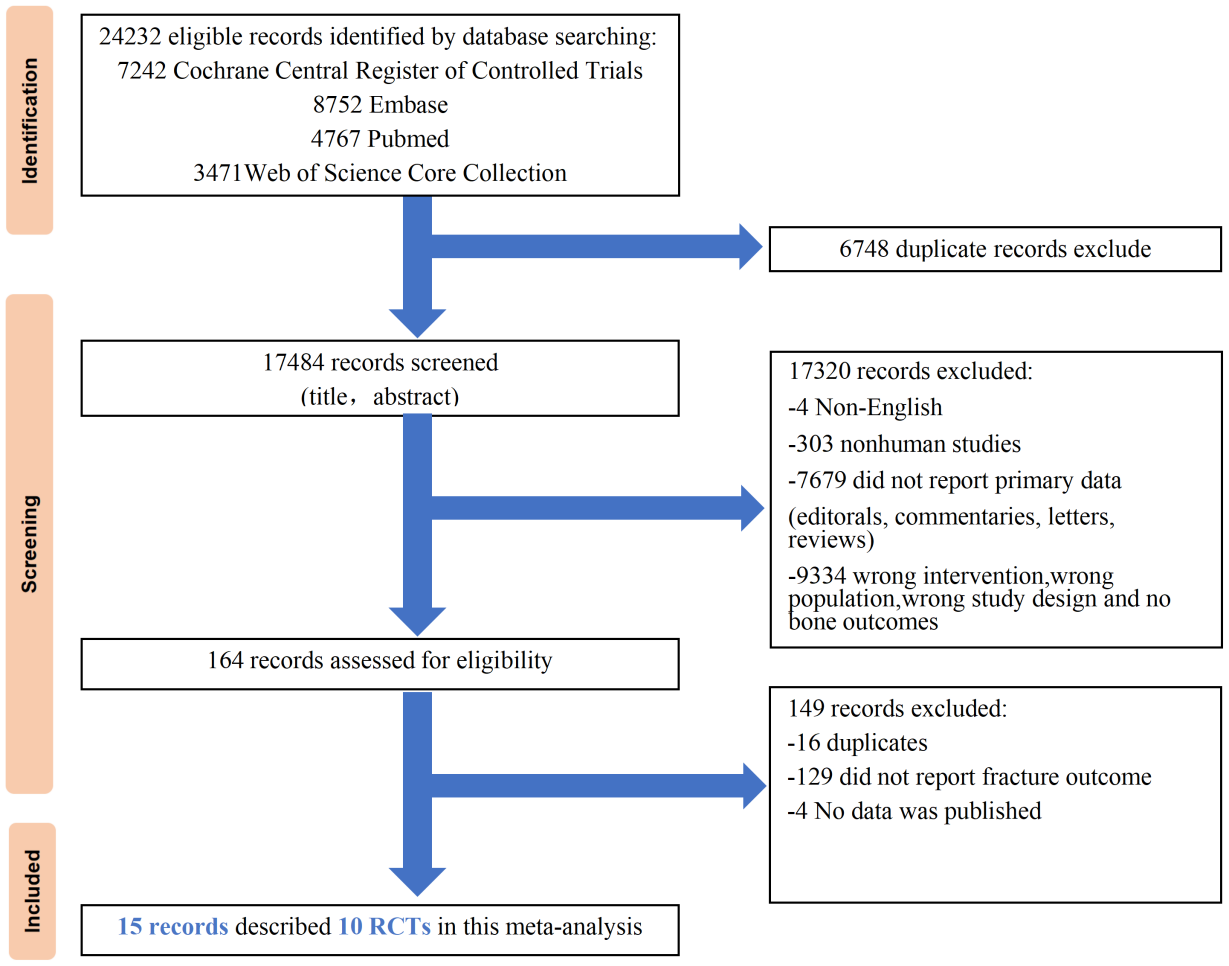
Evidence acquisition flowchart showing results of searches of four databases and screening and final inclusion of studies.

The literature search identified 24,232 records, of which 15 reports corresponding to 10 unique randomized controlled trials (RCTs) met the inclusion criteria (Fig. 1). All included studies investigated the SGLT2i as add-on therapy to insulin in individuals with T1DM. Seven trials employed multiple dose arms, yielding a total of 19 independent treatment comparisons. Six RCTs implemented insulin optimization protocols prior to randomization. Regarding renal function eligibility, three trials excluded patients with impaired renal function (eGFR < 60 ml/min/1.73 m^2^), whereas other seven trials excluded patients with severe renal impairment (eGFR < 30 ml/min/1.73 m^2^). Baseline characteristics were well balanced across treatment arms within each study.

### Studies features

Ten placebo-controlled RCTs comprised 6,731 participants (52% females, mean age 43.2y (SD 2.94)), median diabetic duration 20.5 years, and mean study duration 35.8 weeks (IQR 31.00). Notably, enrolled subjects were relatively young with a mean age of 43.4 (SD 1.72) regardless of cardiovascular risk status. Of the included trials, five RCTs evaluated SOT (Baker et al., 2019; Bode et al., 2021; Buse et al., 2018; Danne et al., 2018; Garg et al., 2017); two RCTs evaluated DAP (Dandona et al., 2018; Mathieu et al., 2020); two RCTs evaluated EMP (Rosenstock et al., 2018) (An article described two RCTs: EASE-2 and EASE-3)(And 1 RCT evaluated IPR Kaku et al., 2019) (Table 2). Besides, seven RCTs performed different SGLT2i dosages subgroup analyses with placebo (Table 2).

**Table 2:** Characteristics of included randomized controlled trials.

Study	Country	N	Follow up (weeks)	Age (years)	Years of T1DM	Female (%)	BMI (kg/m^2^)	HBA1c (%)	Background therapy	Intervention	Control
Baker et al. (2019)	multicenter	141	12	45.6 (13.3)	24.1 (13.7)	73 (51.8)	29.2 (5.6)	8.0 (0.8)	Insulin	SOT 75 mg SOT 200 mg SOT 400 mg	Placebo
Bode et al. (2021)	multicenter	87	12	22.3 (3.8)	11.9 (5.8)	45 (52.9)	28.1 (6.3)	9.8 (1.2)	Insulin	SOT 400 mg	Placebo
Buse et al. (2018)	the U.S. and Canada	793	52	46.1 (13.1)	24.4 (12.8)	410 (51.7)	29.7 (5.4)	7.6 (0.7)	Insulin	SOT 200 mg SOT 400 mg	Placebo
Dandona et al. (2018)	multicenter	833	52	42.4 (13.9)	20.3 (11.8)	405 (52.1)	28.3 (5.4)	8.5 (0.7)	Insulin	DAP 5 mg DAP 10 mg	Placebo
Danne et al. (2018)	multicenter	782	52	41.2 (13.4)	18.4 (10.9)	376 (48.1)	27.8 5.1)	7.8 (0.8)	Insulin	SOT 200 mg SOT 400 mg	Placebo
Garg et al. (2017)	multicenter	1,402	24	42.8(14.1)	20.0(12.3)	705(50.3)	28.2(5.2)	8.2(0.9)	Insulin	SOT 400 mg	Placebo
Kaku et al. (2019)	Japan	175	24	49.2 (12.9)	13.6 (8.6)	93 (53.4)	24.5 (2.9)	8.7 (0.8)	Insulin	IPR 50 mg	Placebo
Mathieu et al. (2020)	multicenter	813	52	42.7 (13.3)	19.3 (11.8)	455 (56.0)	27.6 (5.4)	8.4 (0.7)	Insulin	DAP 5 mg DAP 10 mg	Placebo
Rosenstock et al. (2018) (EASE-3)	multicenter	975	26	43.1 (13.5)	21.0 (12.0)	499 (51.2)	28.3 (5.1)	8.2 (0.6)	Insulin	EMP 2.5 mg EMP 10 mg EMP 25 mg	Placebo
Rosenstock et al. (2018) (EASE-2)	multicenter	730	52	45.1 (13.3)	22.6 (12.7)	385 (53.3)	29.0 (5.4)	8.1 (0.6)	Insulin	EMP 10 mg EMP 25 mg	Placebo

### Assessment on risk of Bias

**Figure 2:**
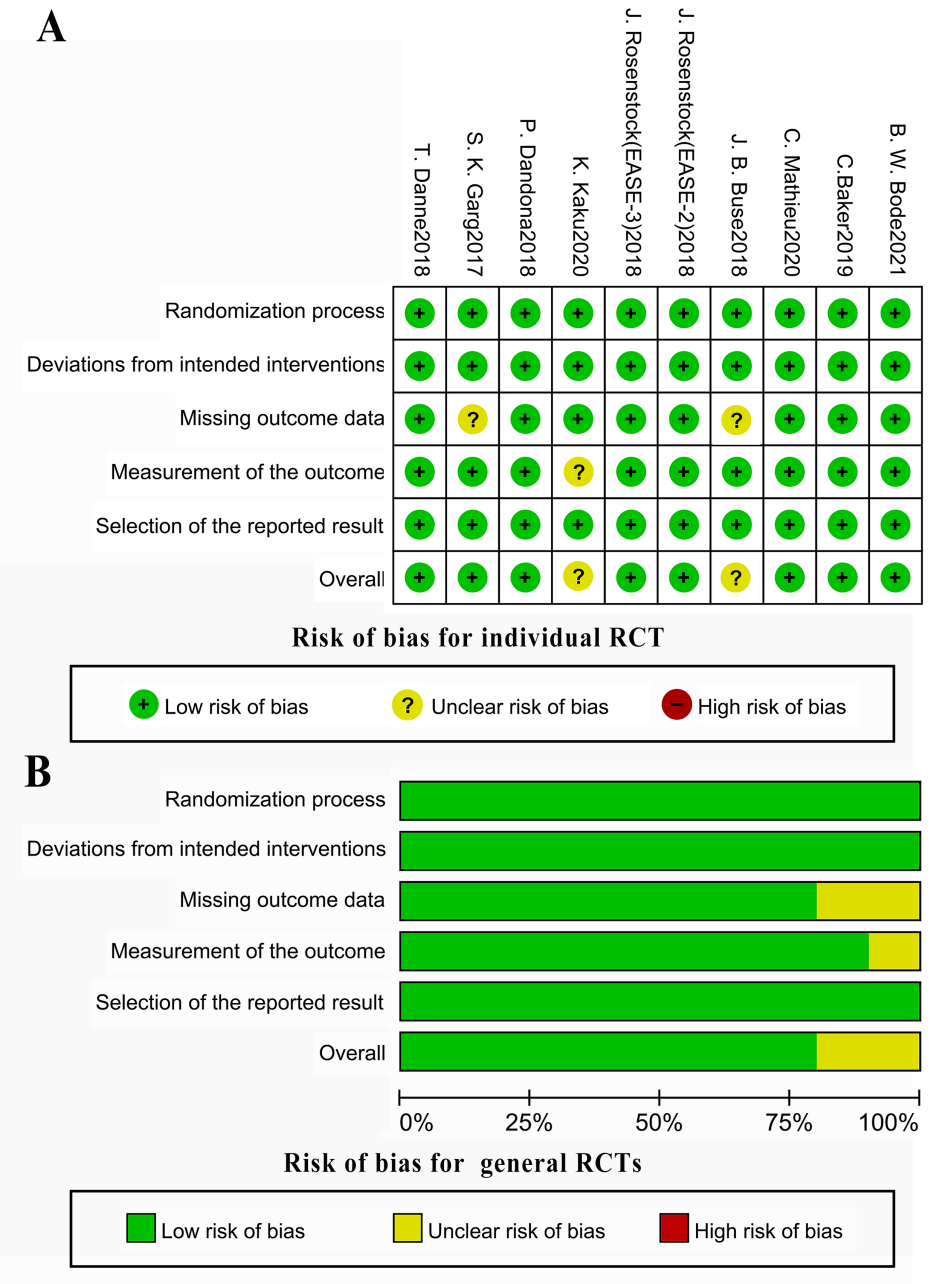
Risk of bias for individual RCT (A) and general RCT (B) were assessed using Cochrane Collaboration Risk-of-Bias (RoB2) tool containing five domains. Ten included RCTs have high quality in five domains (randomization process, deviations from the intended interventions, missing outcomes, measurement of the outcome, and selection of reported results). B plot indicates the proportion of different risks for each domain. Green represents low risk of bias; yellow represents moderate risk of bias; red represents high risk of bias.

The risk of bias for the included RCTs was detailed in Fig. 2. Three RCTs (Buse et al., 2018; Garg et al., 2017; Kaku et al., 2019) had moderate-risk bias in 1 domain but no high risk of sponsorship bias (Fig. 2). Funnel plots of all outcomes were shown in Fig. S1, except for urinary ACR. Visual inspection indicated no substantial publication bias. For urinary ACR, publication bias evaluation was not performed due to limited study numbers. There were three RCTs included in primary analysis and five comparisons included in subgroup analysis in terms of the urinary ACR (Fig. S1).

### Fracture risk

**Figure 3:**
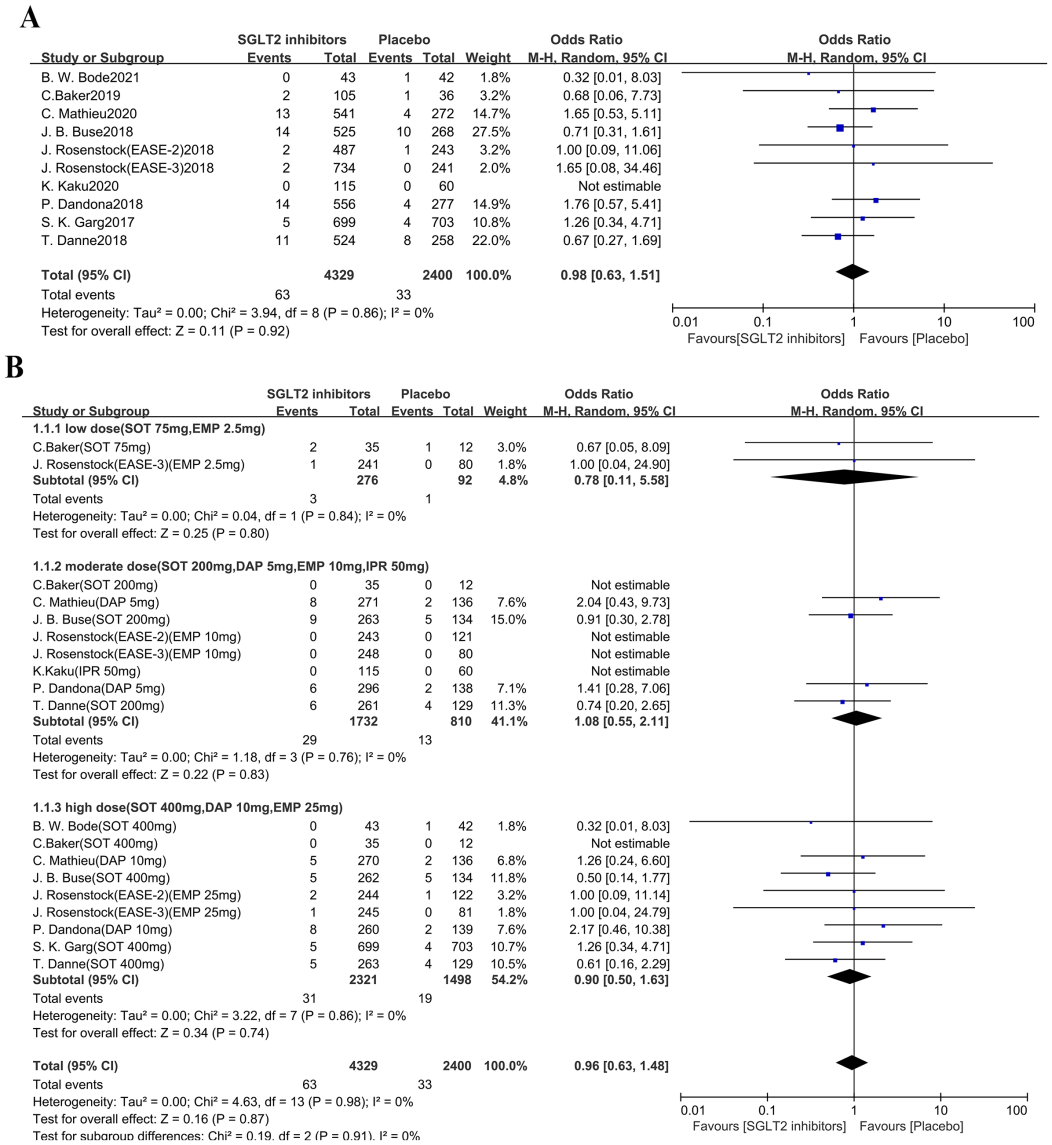
Forest plots to compare the risk of fracture between SGLT2i group and placebo group with insulin therapy in T1DM. Forest plots of primary analysis (A) and subgroup analysis (B) to compare the risk of fracture between SGLT2i group and placebo group with insulin therapy in T1DM. The OR and 95% CI were calculated by the random-effects Mantel-Haenszel method. I^2^ represents the percentage of degree of heterogeneity. OR, Odds Ratio; M-H, Mantel-Haenszel; CI, 95% confidence intervals.

Ten studies (n = 6,729 participants) were involved in primary analysis on fracture risk (Fig. 3). Compared with placebo, SGLT2i had no significant influence on bone fracture when added to insulin therapy in T1DM (OR 0.98, 95% CI [0.63–1.51], *p* = 0.92, I^2^ = 0%). 19 comparisons were included in subgroup analysis based on different dosage of SGLT2i administration. Similarly, no significant association with fracture risk was observed in any dose stratum (Fig. 3). In low dose subgroup, the fracture OR was 0.78 (95% CI [0.11–5.58]; *p* = 0.80, I^2^ = 0%). In moderate dose subgroup, the OR was 1.08 (95% CI [0.55–2.11]; *p* = 0.83, I^2^ = 0%). In high dose subgroup, the OR was 0.90 (95% CI [0.50–1.63]; *p* = 0.74, I^2^ = 0%).

### Glycemic efficacy outcomes

#### Glycosylated hemoglobin type A1C

**Figure 4:**
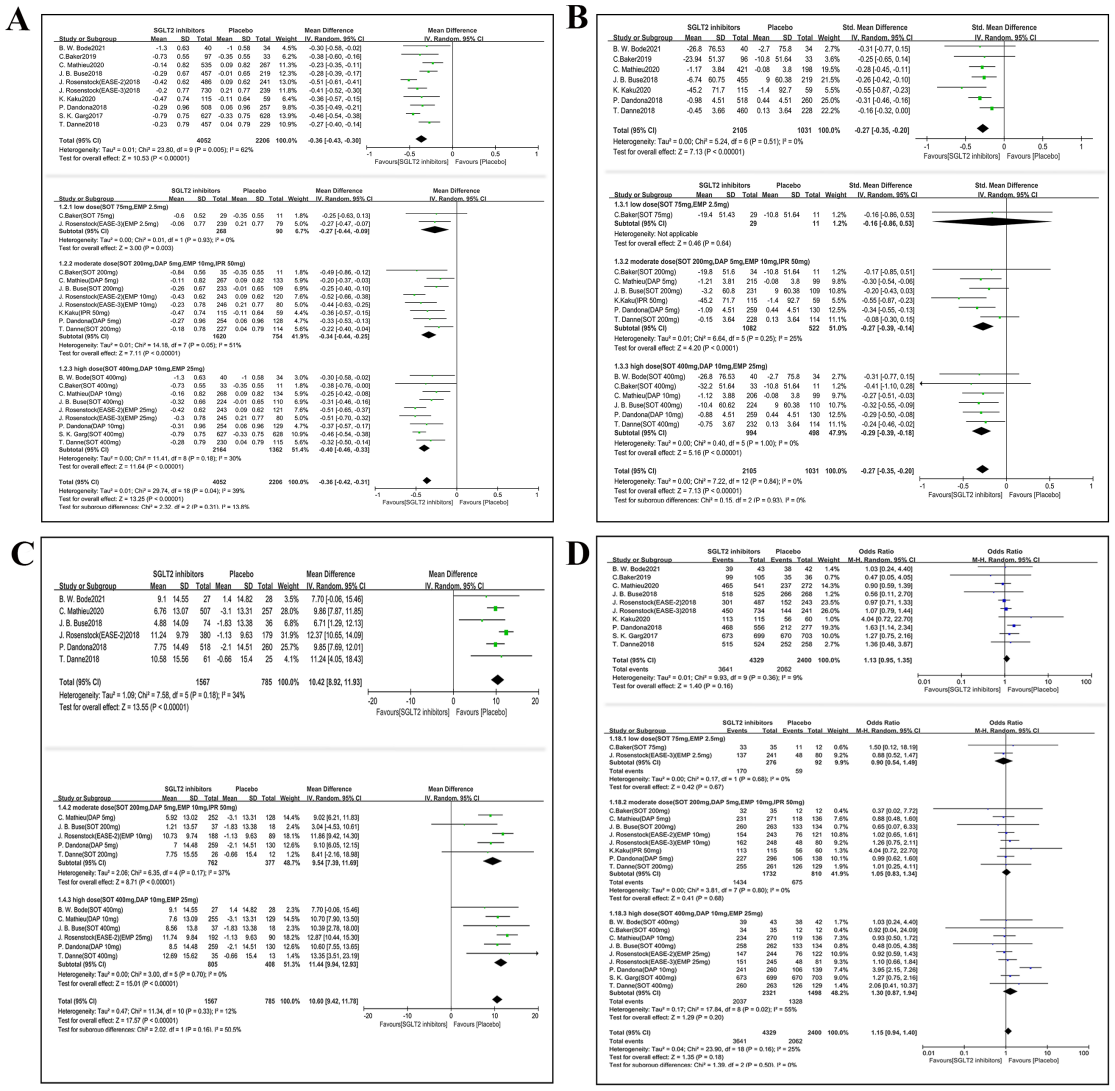
Forest plots indicate the effects of SGLT2i on glycemic efficacy outcomes with insulin therapy in T1DM. The primary analysis is above and the subgroup analysis is below for each plot. (A) HbA1c, (B) Fasting plasma glucose (FPG), (C) Time-in-range (70- 180 mg/dl) (TIR), (D) hypoglycemia. SD, standard deviation; MD, mean average difference; SMD, standardized mean difference; OR, Odds Ratio; M-H, Mantel-Haenszel; CI, 95% confidence intervals.

Ten studies (6,258 participants) reported HbA1c outcome (Fig. 4). Compared with placebo, SGLT2i administration reduced HbA1c level (MD −0.36, 95% CI [−0.43 to −0.30], *p* < 0.001, I^2^ = 62%). A total of 19 comparisons were included in subgroup analyses based on different dosages. It was revealed that the medications had a significantly positive effect on HbA1c in T1DM with background therapy across all dosage subgroups (Fig. 4) (low-dose group: MD −0.27, 95% CI [−0.44 to −0.09], *p* = 0.003, I^2^ = 0%; moderate dose group: MD −0.34, 95% CI [−0.44 to −0.25], *p* < 0.001, I^2^ = 51%; high-dose group: MD −0.40, 95% CI [−0.46 to −0.33], *p* < 0.001, I^2^ = 30%).

#### Fasting plasma glucose

As shown in Fig. 4, seven RCTs (*n* = 3,136 participants) were involved in this part. A total of 13 comparisons were established for subgroup analysis. Compared with placebo, SGLT2i administration was associated with a decrease in level of FPG in T1DM (SMD −0.27, 95% CI [−0.35 to −0.20], *p* < 0.001, I^2^ = 0%). However, there was only one comparison involved in low dose subgroup analysis, showing no statistically significance between medication and control group. Both moderate dosage and high dosage of SGLT2i administration demonstrated significant reductions in FPG (moderate dose group: SMD −0.27, 95% CI [−0.39 to −0.14], *p* < 0.001, I^2^ = 25%; high dose group: SMD −0.29, 95% CI [−0.39 to −0.18], *p* < 0.001, I^2^ = 0%).

#### Time-in-range

Six studies (*n* = 2352 participants) evaluated time in range (TIR) (Fig. 4). Compared with placebo, SGLT2i significantly improved the TIR by 10.42% (MD 10.42, 95% CI [8.92–11.93], *p* < 0.001, I^2^ = 34%). A subgroup analysis of 11 comparisons showed no significant TIR improvement in the low-dose subgroup, which comprised limited data. By contrast, both the moderate-dose (MD 9.54%, 95% CI [7.39–11.69]; *p* < 0.001; I^2^ = 37%) and high-dose (MD 11.44%, 95% CI [9.94–12.93]; *p* <0.001; I^2^ = 0%) subgroups demonstrated significant increases in TIR compared with the control group.

#### Hypoglycemia

The risk of hypoglycemia was reported in ten studies (n = 6,729 participants, Fig. 4). Overall, SGLT2 inhibitor add-on therapy did not significantly alter the risk of hypoglycemia compared with placebo (OR 1.13, 95% CI [0.95–1.35], *p* = 0.16, I^2^ = 9%). Subgroup analysis by dosage (19 comparisons) yielded consistent results. In the low-dose subgroup, the OR of hypoglycemia was 0.90 (95% CI [0.54–1.49]; *p* = 0.67, I^2^ = 0%) In moderate-dose subgroup, the OR was 1.05 (95% CI [0.83–1.34]; *p* = 0.68, I^2^ = 0%). In high-dose subgroup, the OR of hypoglycemia was 1.30 (95% CI [0.87–1.94]; *p* = 0.20, I^2^ = 55%). No significant dose-dependent effect on hypoglycemia risk was observed.

#### Daily total insulin dose

In Fig. S2, TID was depicted in nine studies (*n* = 5,960 participants). Pooled analysis showed that SGLT2i significantly improved TID in our pooled results (SMD −0.56, 95% CI [−0.71 to −0.40], *p* < 0.001, I^2^ = 86%). Dose-stratified analyses (16 comparisons) demonstrated significant reductions across all dosage levels: low-dose group: SMD −0.49, 95% CI [−0.76 to −0.22], *p* < 0.001; moderate dose group: SMD −0.59, 95% CI [−0.82 to −0.35], *p* < 0.001, I^2^ = 85%; high-dose group: SMD −0.55, 95% CI [−0.70 to −0.39], *p* < 0.001, I^2^ = 74%.

However, the overall heterogeneity of primary analysis was high. We found that the studies on ipragliflozin (Kaku et al., 2019) and empagliflozin (Rosenstock et al., 2018) were main contributors to heterogeneity in the moderate dosage subgroup. Two studies on empagliflozin (Rosenstock et al., 2018) (One article described two RCTs: EASE-2 and EASE-3), were the key contributors to heterogeneity in high dose group. Exclusion of these studies attenuated heterogeneity while preserving statistical significance (moderate-dose group: SMD −0.36, 95% CI [−0.48 to −0.23], *p* < 0.001, I^2^ = 25%; high-dose group: SMD −0.44, 95% CI [−0.55 to −0.34], *p* < 0.001, I^2^ = 33%).

#### Daily bolus insulin dose

When it comes to the daily bolus ID change (Fig. S2), 6 studies (3635 participants) were included and nine comparisons were involved in the subgroup analysis. Compared with placebo, a decrease in the daily bolus ID was evident (SMD −0.26, 95% CI [−0.37 to −0.14], *p* < 0.001, I^2^ = 56%). Significant reductions persisted in dose-stratified analyses (moderate dose group: SMD −0.29, 95% CI [−0.50 to −0.08], *p* < 0.05, I^2^ = 68%; high dose group: SMD −0.23, 95% CI [−0.31 to −0.15], *p* < 0.001, I^2^ = 0%). Sensitivity analysis identified one ipragliflozin comparison (Kaku et al., 2019) as a major source of heterogeneity in the moderate-dose subgroup. With its exclusion, the effects remained statistically significant and the heterogeneity was reduced. After this adjustment, the SMD in moderate dose group became to −0.19 (95% CI [−0.32 to −0.06], *p* < 0.05, I^2^ = 0%).

#### Daily basal insulin dose

Basal insulin stands for the application of long-acting insulin analog to control basal blood glucose (Horton, 2009). It was regarded as an important parameter to assess the effectiveness of antidiabetic therapy. Six RCTs (3,654 participants) reported on the analysis of daily basal insulin dose changes (Fig. S2). Nine comparisons were included in the subgroup analyses. Compared with placebo, SGLT2i decreased the daily basal insulin uasge (SMD −0.50, 95% CI [−0.69 to −0.31], *p* < 0.001, I^2^ = 84%). Subgroup analysis also showed significant reductions in moderate dose group (SMD −0.55, 95% CI [−0.87 to −0.23], *p* < 0.001, I^2^ = 86%) and high dose group (SMD −0.45, 95% CI [−0.64 to −0.27], *p* < 0.001, I^2^ = 72%). To address this heterogeneity, sensitivity analyses were performed. After removing the ipragliflozin study (Kaku et al., 2019) from the moderate-dose group (SMD −0.38, 95% CI [−0.51 to −0.24], *p* < 0.001, I^2^ = 12%), and the dapagliflozin study (Mathieu et al., 2020) from the high-dose group (SMD −0.38, 95% CI [−0.51 to −0.25], *p* < 0.001, I^2^ = 31%), the results remained significant.

### Nonglycemic efficacy outcomes

#### Body weight changes

**Figure 5:**
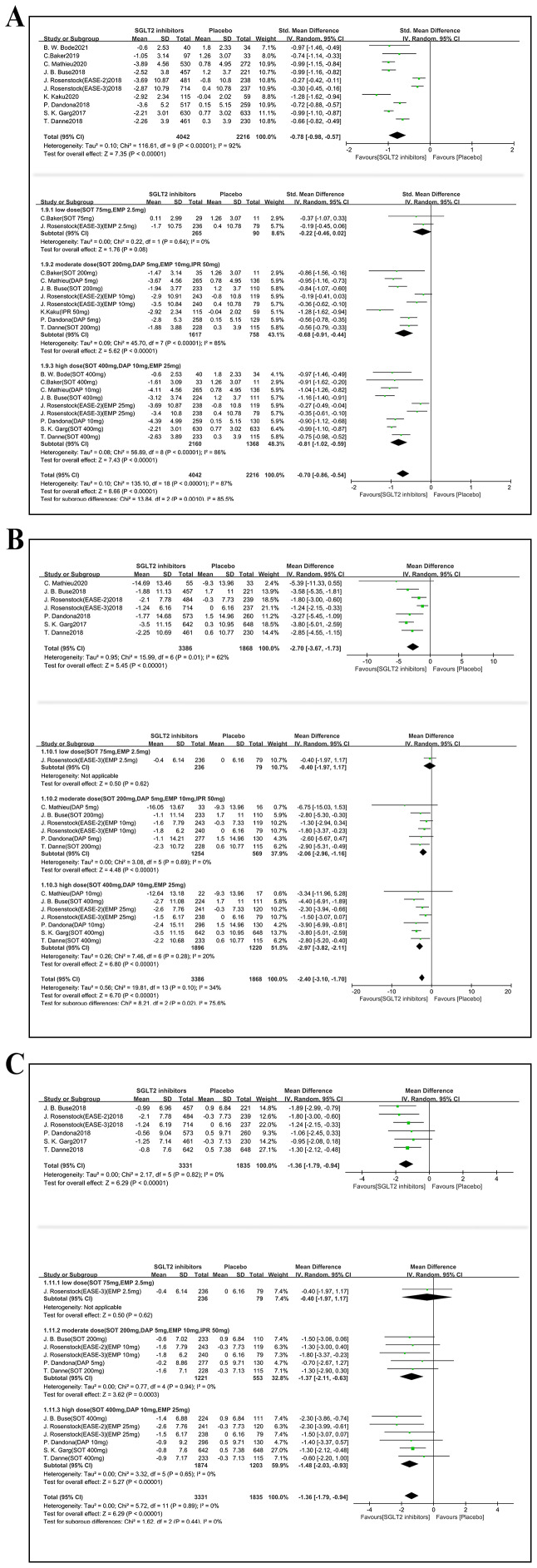
Forest plots show the effects of SGLT2i on nonglycemic efficacy outcomes with insulin therapy in T1DM. The primary analysis is above and the subgroup analysis is below for each plot. Outcomes: (A) Body weight changes, (B) Systolic blood pressure (SysBP), (C) Diastolic blood pressure (DBP). SD, Standard deviation; MD, mean average difference SMD, standardized mean difference: M-H, Mantel-Haenszel; CI, 95% confidence intervals.

In Fig. 5, body weight change was evaluated in ten studies (n = 6,258 participants). Compared with placebo, the SGLT2i group had a lower body weight (SMD −0.78, 95% CI [−0.98 to −0.57], *p* < 0.001, I^2^ = 92%). Subgroup analyses included 19 comparisons. Significant positive effects on body weight were identified in the moderate-dose and high-dose groups. In contrast, only two comparisons included in the low-dose group, and they showed no significant effect (low-dose group: SMD −0.22, 95% CI [−0.46–0.02], *p* = 0.08, I^2^ = 0%; moderate-dose group: SMD −0.68, 95% CI [−0.91 to −0.44], *p* < 0.001, I^2^ = 85%; high-dose group: SMD −0.81, 95% CI [−1.02 to −0.59], *p* < 0.001, I^2^ = 86%).

Obviously, substantial heterogeneity among studies in subgroup comparisons was high. Three articles related to ipragliflozin (Kaku et al., 2019) and empagliflozin (Rosenstock et al., 2018) in moderate dose group and two studies on empagliflozin (Rosenstock et al., 2018) in high dose group were recognized as the source of heterogeneity. After excluding these studies, the effects remained statistically significant with reduced heterogeneity (moderate dose group: SMD −0.73, 95% CI [−0.91 to −0.55], *p* < 0.001, I^2^ = 57%; high dose group: SMD −0.97, 95% CI [−1.06 to −0.88], *p* < 0.001, I^2^ = 9%).

#### Blood pressure changes

As for the influence on blood pressure (Fig. 5), SysBP was reported in seven studies (n = 5,254 participants). A decrease in SysBP was discovered in experimental group (MD −2.70, 95% CI [−3.67, −1.73], *p* < 0.001, I^2^ = 62%). Consistently, subgroup analysis across 15 comparisons revealed that the treatment decreased SysBP. Details were displayed as follows: MD in moderate-dose subgroup was −2.06, with 95% CI [−2.96 to −1.16] (*p* < 0.001, I^2^ = 0%); MD in high dose subgroup was −2.97, with 95% CI [−3.82 to −2.11] (*p* < 0.001, I^2^ = 20%).

In terms of DBP (Fig. 5), six studies (5,166 participants, involving 12 comparisons) were enrolled in our study. Compared with placebo group, SGLT2i, as an add-on to insulin therapy, lowered the DBP level in T1DM patients (MD −1.36, 95% CI [−1.79 to −0.94], *p* < 0.001, I^2^ = 0%). Similarly, these effects were statistically significant in subgroup analyses (moderate-dose group: MD −1.37, 95% CI [−2.11 to −0.63], *p* < 0.001, I^2^ = 0%; high-dose group: MD −1.48, 95% CI [−2.03 to −0.93], *p* < 0.001, I^2^ = 0%).

#### Renal effects: estimated glomerular filtration rate and urinary albumin to creatinine ratio

Five studies contributed to the analysis of estimated eGFR, comprising nine comparisons (n = 2,820 patients) (Fig. S3). No beneficial effect of SGLT2i on eGFR was identified. SMD of eGFR was −0.07, with 95% CI [−0.15–0.01], (*p* = 0.07, I^2^ = 0%). There was one comparison in low-dose subgroup, which showed no statistically significant effects of the medication. In moderate-dose group, the SMD was −0.15(95% CI [−0.31–0.01], *p* = 0.06, I^2^ = 0%); In high-dose group, the SMD was −0.05(95% CI [−0.13–0.04], *p* = 0.31, I^2^ = 0%).

For urinary ACR, three studies (2,638 patients), consisting of five comparisons were included (Fig. S3). No statistically significant effects of SGLT2i on urinary ACR were observed in the pooled analysis (MD −11.53 95% CI [−27.11–4.05], *p* = 0.15, I^2^ = 18%) and subgroup analyses (moderate-dose group: MD −3.14, 95% CI [−31.01–24.72], *p* = 0.82, I^2^ = 27%; high-dose group: MD −16.15, 95% CI [-33.67–1.38], *p* = 0.07, I^2^ = 0%).

#### Diabetic eye disorders

Five studies involving 10 comparisons (4722 participants) were analyzed for diabetic eye disorders (Fig. S3). As a result, SGLT2i did not affect the occurrence of diabetic eye disorders (OR 0.47, 95% CI [0.15–1.45], *p* = 0.19, I^2^ = 6%). Similar results were detected in subgroup analyses. There was one comparison in low-dose subgroup, which showed no statistical significance between groups. In moderate-dose group, the OR was 0.51 (95% CI [0.09–2.94], *p* = 0.45, I^2^ = 27%); In high-dose group, the OR was 0.36 (95% CI [0.08–1.61], (*p* = 0.18, I^2^ = 0%). Thus, SGLT2i did not significantly alter the risk of diabetic eye disorders across all dosage subgroups.

**Figure 6:**
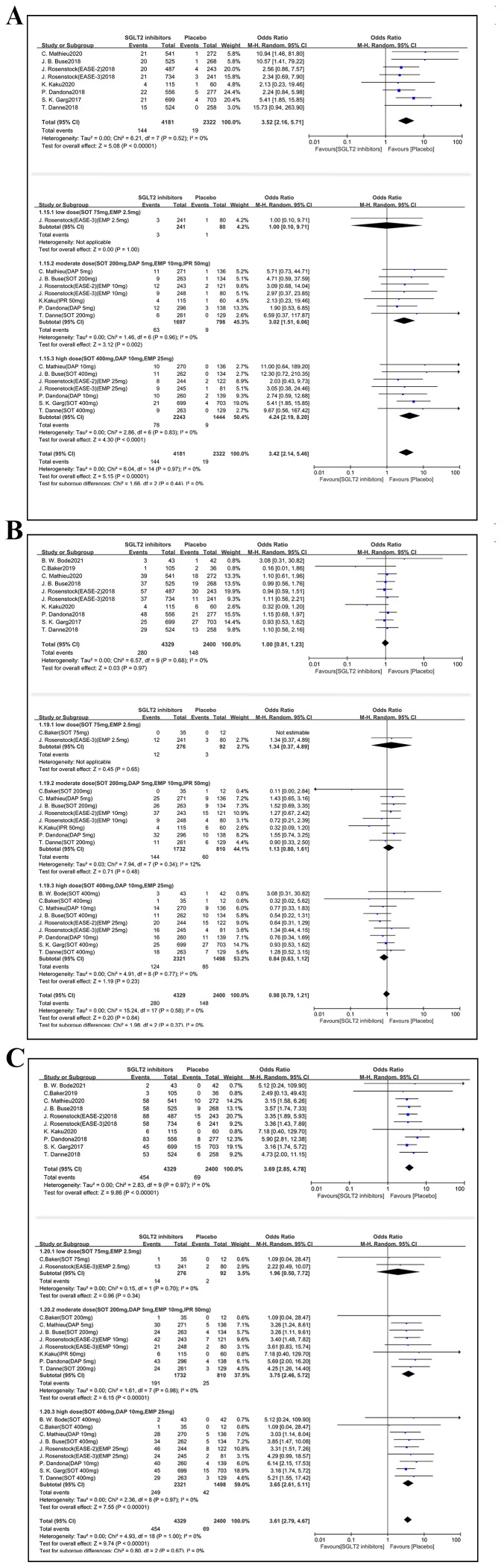
Forest plots indicate the influence of SGLT2i on safety outcomes added to insulin therapy in T1DM. The primary analysis is above and the subgroup analysis is below for each plot. (A) Diabetic ketoacidosis (DKA), (B) Urinary tract infections (UTIs), (C) Genital tract infections (GTIs). OR, Odds Ratio; M-H, Mantel-Haenszel; CI, 95% confidence intervals.

### Other safety outcomes

#### Diabetic ketoacidosis

Eight studies (6,503 participants) were involved in our analysis (Fig. 6). In subgroup analysis, 15 comparisons were extracted. Overall, treatment with SGLT2i raised the risk of DKA in patients with T1DM compared to placebo (OR 3.52, 95% CI [2.16–5.71], *p* < 0.001, I^2^ = 0%). Because there was only one comparison regarding empagliflozin in low dose subgroup analysis, this limited comparison showed no statistically significant results. In contrast, in the moderate and high dosage comparisons, SGLT2i increased the risk of DKA (moderate dose group: OR 3.02, 95% CI [1.51–6.06], *p* < 0.05, I^2^ = 0%; high dose group: OR 4.24, 95% CI [2.19–8.20], *p* < 0.001, I^2^ = 0%).

#### Cardiovascular events

Eight studies (6,503 participants) were enrolled in the analysis of the effect of SGLT2i on major adverse cardiovascular events (MACE) risk (Fig. S3). Subgroup analysis comprised 15 comparisons. As a result, SGLT2i add-on therapy had no effect on the risk of MACE (OR 0.99, 95% CI [0.52–1.89], *p* = 0.99, I^2^ = 0%), either in primary or subgroup analyses.

#### Urinary tract infections

Ten studies involving 6,729 participants contributed data on UTIs (*n* = 6,729 participants) (Fig. 6). Overall, no significant association was observed between SGLT2i use and the risk of UTIs (OR 1.00, 95% CI [0.81–1.23], *p* = 0.97, I^2^ = 0%). Subgroup analyses were performed based on dosage across 19 comparisons, and the results remained consistent with the primary analysis (low dose group: OR 1.34, 95% CI [0.37–4.89], *p* = 0.65; moderate dose group: OR 1.13, 95% CI [0.80–1.61], *p* = 0.48, I^2^ = 12%; high dose group: OR 0.84, 95% CI [0.63–1.12], *p* = 0.23, I^2^ = 0%).

#### Genital tract infections

Ten studies (*n* = 6729 subjects) reported data on GTIs (Fig. 6). SGLT2i therapy in conjunction with insulin therapy conferred a significantly increased risk of GTIs (OR 3.69, 95% CI [2.85–4.78], *p* < 0.001, I^2^ = 0%). Subgroup analyses were conducted across 19 comparisons. No significant association was found in low dose subgroup, which included 2 comparisons (OR 1.96, 95% CI [0.50–7.72], *p* = 0.34, I^2^ = 0%). However, both moderate and high dosage of SGLT2i significantly elevated GTIs risks (moderate dose group OR 3.75, 95% CI [2.46–5.72], *p* < 0.001, I^2^ = 0%; high dose group OR 3.65, 95% CI [2.61–5.11], *p* < 0.001, I^2^ = 0%).

### Sensitivity analysis

We performed sensitivity analyses to assess the robustness of our findings. First, switching from a random-effects to a fixed-effects model did not alter the statistical significance or direction of any pooled result. Second, leave-one-out analysis revealed that only three outcomes (eGFR, urinary ACR, and diabetic eye disorders) changed from non-significant to significant after removal of individual study (Buse et al., 2018; Dandona et al., 2018; Garg et al., 2017). These outcomes were based on limited numbers of trials (eGFR: five studies; UACR: three studies; diabetic eye disorders: five studies), indicating low statistical stability and susceptibility to individual study influence. All other outcomes remained robust statistical significance across sensitivity analyses (Table S2).

### Grading of evidence

**Figure 7:**
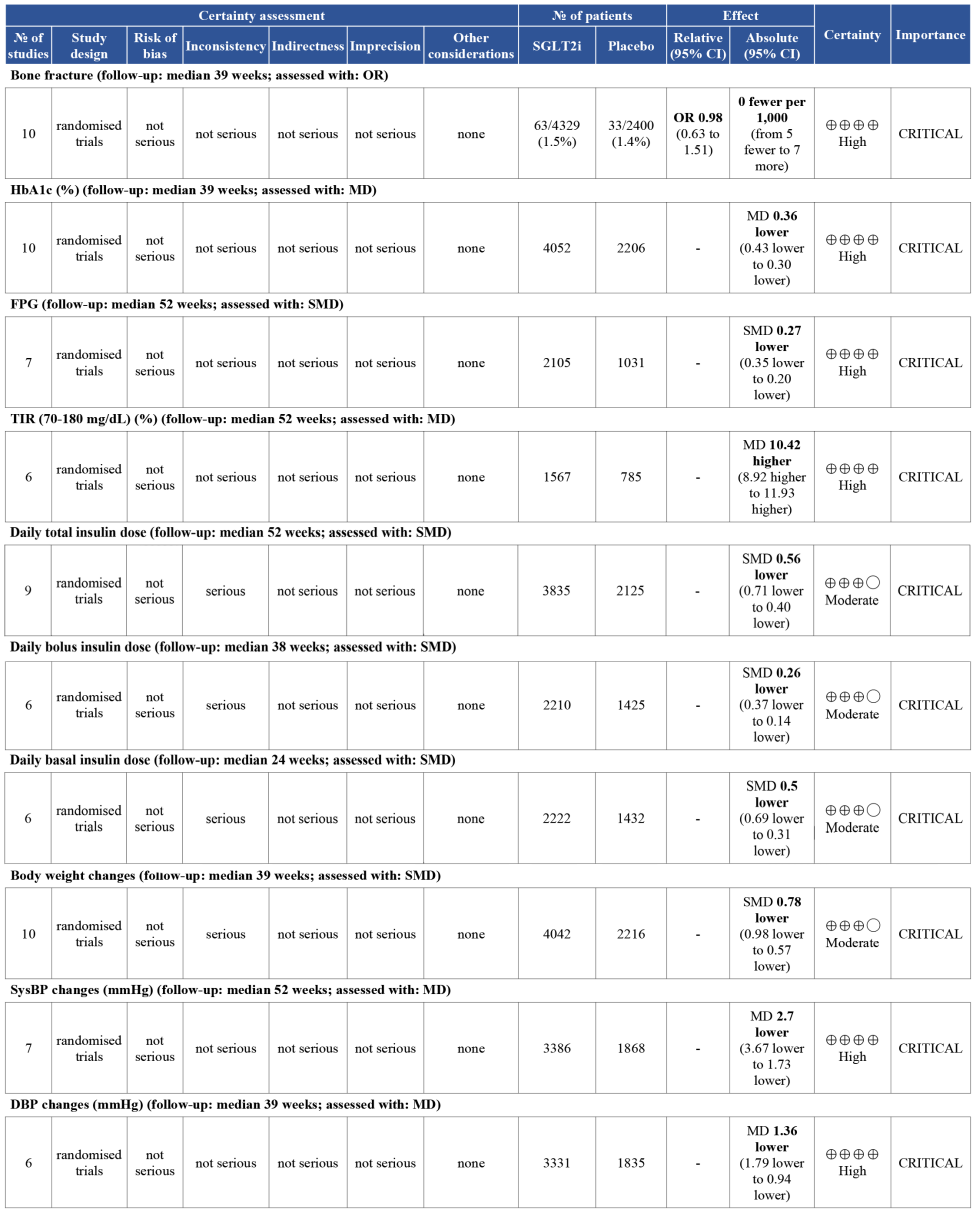
The certainty of the evidence of ten outcomes by using the Grading of Recommendations Assessment, Development, and Evaluation (GRADE) approach. Outcomes: bone fracture, HbA1c, Fasting plasma glucose (FPG), Time-in-range (70–180 mg/dl) (TIR), Daily total insulin dose, Daily bolus insulin dose, Daily basal insulin dose, Body weight changes, Systolic blood pressure (SysBP), Diastolic blood pressure (DBP).

**Figure 8:**
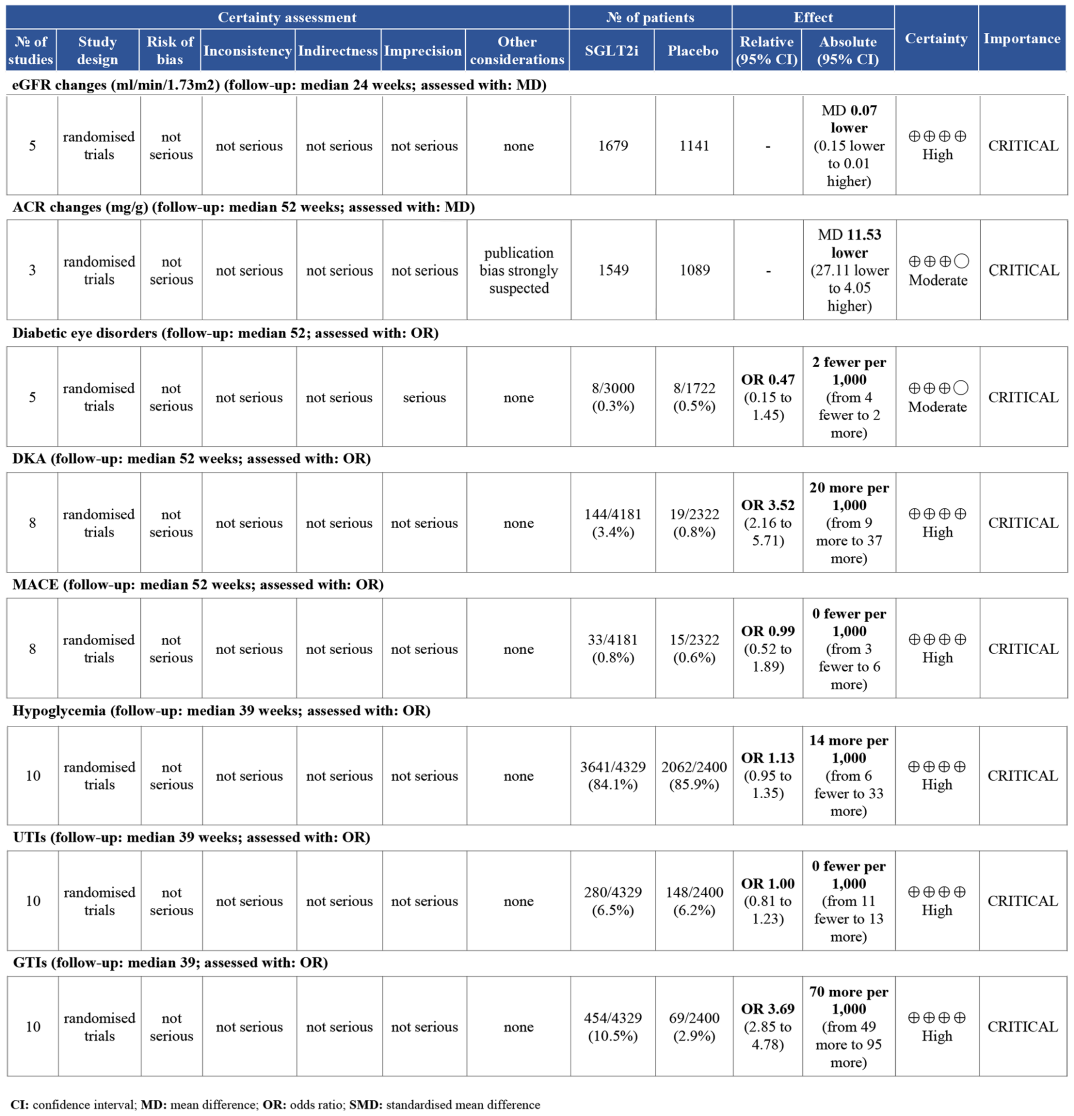
The other certainty of the evidence of eight outcomes by using the Grading of Recommendations Assessment, Development, and Evaluation (GRADE) approach. Outcomes: estimated glomerular filtration rate (eGFR), urinary albumin to creatinine ratio (ACR), diabetic eye disorders, diabetic ketoacidosis (DKA), Cardiovascular events (MACE), hypoglycemia, Urinary tract infections (UTIs), Genital tract infections (GTIs).

The quality of the evidence was shown in Figs. 7 and 8. Four outcomes (daily total insulin dose, daily bolus insulin dose, daily basal insulin dose, and body weight) were downgraded to meet the moderate quality due to substantial heterogeneity. Regrettably, publication bias of urinary ACR could not be assessed due to the limited studies included in this analysis. The quality of the evidence was also downgraded to a moderate level. No outcomes were rated as low or very low certainty.

## Discussion

Due to insulin deficiency, patients with T1DM rely on insulin therapy to achieve glucemic control. However, many continued to experience suboptimal glucose variability and suboptimal treatment adherence. Adjunctive oral agents may therefore offer a promising strategy to enhance glycemic stability. SGLT2i represented a novel therapeutic class that reduced hyperglycemia through insulin-independent enhancement of urinary glucose excretion. Notably, prior investigations in T2DM populations have yielded conflicting evidence regarding the skeletal safety of these agents—some studies suggesting raised risk of fracture (Sridharan & Sivaramakrishnan, 2025), while others found no such association (Donnan et al., 2019; Van Hulten et al., 2021; Wang et al., 2023). The present meta-analysis specifically addresses this evidence gap by evaluating the effect of SGLT2i on fracture risk in insulin-treated patients with T1DM.

Our principal finding indicated that adjunctive SGLT2i therapy does not significantly increase fracture risk in this population. Furthermore, SGLT2i significantly improved key glycemic control (HbA1c, fasting plasma glucose, time in range) and reduced daily insulin requirements. Also, it improved the blood pressure and promoted weight loss. Notably, application of SGLT2i had no risk of hypoglycemia and UTIs. However, it was associated with the elevated risk of DKA and GTIs. The absence of demonstrable improvements in cardiovascular and renal outcomes likely reflected the relatively young age of enrolled participants and the limited number of available RCTs with adequate follow-up duration. Collectively, these findings suggested that SGLT2i had a positive effect on stable glucose control in patients with T1DM, without increasing fracture risk, but concerns of inscreased risk of DKA and GTIs.

Our meta-analysis primarily assessed the effect of SGLT2 inhibitor add-on therapy on fracture risk in patients with type 1 diabetes receiving insulin. Data from 6,729 participants showed that SGLT2i did not significantly alter fracture risk compared with placebo. Mechanistically, SGLT2 inhibition may influence bone metabolism through renal effects on electrolyte and mineral homeostasis. By increasing tubular phosphate reabsorption, SGLT2i can elevate serum phosphate, which in turn stimulates parathyroid hormone (PTH) secretion (Taylor, Blau & Rother, 2015). This cascade may promote fibroblast growth factor-23 (FGF-23) release from osteocytes and reduce 1,25-dihydroxyvitamin D levels—both pathways implicated in bone turnover and potentially in fracture susceptibility. Nonetheless, our pooled clinical data did not translate these mechanistic possibilities into a detectable increase in fracture incidence in this population.

In contrast, our study identified no effect of SGLT2i on fracture risk in T1DM patients receiving insulin therapy. Several factors may account for this null finding. The enrolled population exhibited a mean age of 43.2 years (SD 2.94), with a long-standing diabetes (median duration of 20.53 years), representing a cohort at relatively low baseline risk of fragility fractures. Moreover, the mean treatment duration was just 35.8 weeks, reflecting relatively short-term exposure to SGLT2i (IQR 31.00). Thus, the confounding factors of age, diabetes duration, and therapy duration cannot be overlooked. Unfortunately, most of the involved RCTs did not report key bone-metabolism biomarkers, such as calcium, phosphate and BMD. Consequently, we speculated that the limited indicators could not entirely reflect the exact role of SGLT2i in bone metabolism in T1DM, resulting in reduced grades of evidence. Further studies should incorporate comprehensive bone-related enpoints to clarify the long-term skeletal safety of SGLT2i in T1DM.

As the supplementary treatment of SGLT2i combined with insulin, it improved glycemic control, reduced both blood pressure and promoted weight loss. These results were consistent with previous findings (Biester et al., 2021; Katsimardou et al., 2024; Nan et al., 2024; Powell et al., 2020). However, the absence of a significant effect on time-in-range in the low-dose group in our study should be interpreted with caution. Importantly, SGLT2i raised the risk of DKA (OR:3.52), which was consistent with the previous evidence (Li & Xu, 2019). The mean BMI of the pooled population was 28.29 kg/m^2^ (SD 0.88), indicating that most participants were overweight. Traditionally, low BMI was regarded as a risk factor for DKA in T1DM. The elevated DKA risk associated with SGLT2i was identified in our study as well. This risk was also confirmed in patients with T2DM after SGLT2i medication (Liu et al., 2020). A large meta-analysis of 39 RCTs indicated that the use of SGLT2i in T2DM was associated with an increased risk of DKA (OR 2.13) (Liu et al., 2020). We hypothesized that the underlying insulin deficiency characteristic of type 1 diabetes may amplify this risk through complex metabolic interactions.

SGLT2i use was associated with an elevated risk of GTIs (OR 3.69), a finding consistent with prior literature (Kamei & Yamamoto, 2021). Although the low-dose subgroup (only two comparisons) showed no significant effect, moderate- and high-dose subgroups both demonstrated increased GTI risk. Mechanistically, glycosuria induced by SGLT2 inhibition raised genital glucose concentrations, creating a favorable environment for yeast overgrowth (Fralick & MacFadden, 2020). In contrast, we observed no significant increase in UTI risk with SGLT2i. This may be explained by continuous urinary flushing, which could limit the accumulation of glucose metabolites in the urinary tract. Similarly, real-world studies have reported no clinically significant rise in UTI risk with SGLT2i (Wiegley & So, 2022). However, evidence remained conflicting: some studies suggested a dose-dependent increase in UTI risk with specific agents such as dapagliflozin (Geerlings et al., 2014), while others reported no association (Dave et al., 2019; Iordan et al., 2024). Thus, the impact of SGLT2i on UTI risk remained inconclusive.

Interestingly, there was no significant influence of SGLT2i on eGFR and urinary ACR. Most participants had normal or only mildly impaired renal function at baseline, without overt diabetic nephropathy. It was plausible that the renoprotective benefits of SGLT2i were more pronounced in patients with established renal dysfunction (Sloan, 2024), and the relatively short observation periods in the included trials may have limited the detection of such effects. SGLT2i exerted osmotic diuretic actions that can reduce the volume overload, lower the blood pressure, and potentially preserve the cardiac function (Vallon & Verma, 2021). In patients with type 2 diabetes at high cardiovascular risk, these agents have been shown to improve cardiovascular outcomes (Marilly et al., 2022). In contrast, our meta-analysis did not identify a significant reduction in major adverse cardiovascular events with SGLT2i in type 1 diabetes. This may be attributable to the younger age of the included population (mean age 43.4 years) and the relatively short follow-up durations, which likely limited the accrual of cardiovascular endpoints.

Finally, we also used the leave-one-out sensitivity analysis to see if any of the studies significantly impacted the results. Notably, three outcomes (eGFR, ACR, and Diabetic eye disorders) became meaningful after removing a single trial in each case. However, these outcomes were based on a limited number of studies (eGFR: five trials; UACR: three trials; diabetic eye disorders: five trials), which reduced the stability of the results. Furthermore, formal assessment of publication bias was not feasible for UACR due to the small number of included studies, leading to a downgrade in the quality of evidence for this outcome. In summary, the current evidence regarding the effects of SGLT2i on eGFR, UACR, and diabetic eye disorders in type 1 diabetes remained inconclusive and sensitive to individual study data. Further high-quality trials with larger sample sizes and longer follow-up were needed to clarify their impact on these endpoints.

## Conclusions

In conclusion, SGLT2i combined with insulin therapy improved glycemic control in patients with TIDM without raising fracture risk. Our analysis also found no elevated risk of hypoglycemia or diabetic eye disorders with SGLT2i. Possibly limited by the baseline characteristics of the included population, this study did not find an improvement in cardiac and renal outcomes in T1DM patients using SGLT2i. Importantly, SGLT2i were associated with increased risks of diabetic ketoacidosis and genital tract infections, which represented important safety considerations in clinical practice. Further studies with longer follow-up and broader patient profiles were needed to clarify the long-term cardiorenal effects and safety profile of SGLT2i in this specific population.

## Limitation

Our meta-analysis had several limitations. Since SGLT2i are not widely used for T1DM currently, there are limited clinical RCTs meeting our inclusion criteria. Fracture risk and bone metabolism alterations typically require long-term observation. However, the included studies were of relatively short duration and involved predominantly younger patients, which may explain the absence of a significant fracture signal. Furthermore, fracture was not the primary endpoint for most studies, potentially limiting the completeness and accuracy of fracture reporting. These factors underscore the need for longer-term, prospectively designed studies with bone-focused outcomes to fully assess the skeletal safety of SGLT2i as adjunctive therapy to insulin in T1DM.

## Supplemental Information

10.7717/peerj.21087/supp-1 Supplemental Information 1 Detailed strategies for searching the four databases

10.7717/peerj.21087/supp-2 Supplemental Information 2 Sensitivity analysis (Mantel-Haenszel method, fixed-effect model, and Leave-one-out sensitivity analysis)

10.7717/peerj.21087/supp-3 Supplemental Information 3 Funnel plots of comparisons show publication bias for the outcomes Publication bias for the outcomes. Outcomes: (A) fracture risk, (B) HbA1c, (C) Fasting plasma glucose ( FPG), (D) Time-in-range (70-180 mg/dl) (TIR), (E) Daily total insulin dose, (F) Daily bolus insulin dose, (G) Daily basal insulin dose, (H) Body weight changes, (I) Systolic b lood p ressure (SysBP), (J) Diastolic blood pressure (DBP), (K) Estimated glomerular filtration rate (eGFR), (L) Diabetic eye disorders, (M) Diabetic ketoacidosis (DKA), (N) Cardiovascular events (MACE), (O) Hypoglycemia, (P) Urinary tract infections (UTIs), (Q) Genital tract infections (GTIs). SE, standard error; MD, mean difference; SMD, standardized mean difference: OR, odds ratio.

10.7717/peerj.21087/supp-4 Supplemental Information 4 Forest plots indicate the effects of SGLT2i on three glycemic outcomes adding to insulin therapy in T1DM The effects of SGLT2i on glycemic efficacy outcomes with insulin therapy in T1DM. The primary analysis is above and the subgroup analysis is below for each plot. (A) Daily t otal insulin dose, (B) Daily bolus insulin dose, (C) Daily basal insulin dose, (D) hypoglycemia. SD, standard deviation; MD, mean average difference; SMD, standardized mean difference; M-H, Mantel-Haenszel; CI, 95% confidence intervals.

10.7717/peerj.21087/supp-5 Supplemental Information 5 Forest plots indicate the effects of SGLT2i on four outcomes adding to insulin therapy in T1DM, compared with placebo group The effects of SGLT2i on four outcomes adding to insulin therapy in T1DM, compared with placebo group. The primary analysis is above and the subgroup analysis is below for each plot. (A) estimated glomerular filtration rate (eGFR), (B) urinary albumin to creatinine ratio (ACR), (C) diabetic eye disorders, (D) Cardiovascular events (MACE). SD, Standard deviation; MD, mean average difference; SMD, standardized mean difference; OR, Odds Ratio; M-H, Mantel-Haenszel; CI, 95% confidence intervals.

10.7717/peerj.21087/supp-6 Supplemental Information 6 Raw data

10.7717/peerj.21087/supp-7 Supplemental Information 7 PRISMA checklist

## Additional Information and Declarations

### Data Availability

The following information was supplied regarding data availability: This is a Systematic Review/Meta-analysis.
