# Supplementary material for: Effect of sodium-glucose cotransporter-2 inhibitors on fracture risk in patients with type 1 diabetes receiving insulin-based therapy: a meta-analysis
Source: PeerJ. 2026 Apr 16;14:e21087. doi: 10.7717/peerj.21087 (PMC13092229; doi:10.7717/peerj.21087)
Supplement: Supplemental Information 1 [file peerj-14-21087-s001.doc]

**Supplementary material 1. Detailed strategies for searching the four databases.**

| **Operator** | **Concept** | **Search terms** |
| --- | --- | --- |
| **Pubmed (4767)** | | |
|  | RCT strategy | (randomized controlled trial [Publication Type] OR controlled clinical trial [Publication Type] OR randomized [Text Word] OR clinical trial as topic [MeSH Terms] OR randomly [Title/Abstract] OR trial [Title] OR placebo [Text Word]) |
| AND | SGLT2i treatment | (sodium glucose transporter 2 [MeSH Terms] OR sodium glucose transporter 2 [Text Word] OR SGLT2 [Text Word] SGLT-2 [Text Word] OR Dapagliflozin [Text Word] OR Farxiga [Text Word] OR BMS-512148 [Text Word] OR Canagliflozin [Text Word] OR Invokana [Text Word] OR JNJ-28431754 [Text Word] OR Empagliflozin [Text Word] OR Jardiance [Text Word] OR BI 10773 [Text Word] OR Ipragliflozin [Text Word] OR Suglat [Text Word] OR ASP1941 [Text Word] OR Luseogliflozin [Text Word] OR Lusefi [Text Word] OR TS-071 [Text Word] OR Tofogliflozin [Text Word] OR Apleway [Text Word] OR Deberza [Text Word] OR Ertugliflozin [Text Word] OR PF04971729 [Text Word] OR MK-8835 [Text Word] OR Sotagliflozin [Text Word] OR LX4211 [Text Word] OR Zynquista [Text Word] OR Bexagliflozn [Text Word] OR EGT0001442 [Text Word] OR Remogliflozin [Text Word] OR remogliflozin etabonate [Text Word] OR GSK 189075 [Text Word] OR ISIS 388626 [Text Word] OR ISIS-SGLT2Rx [Text Word] OR TA-7284 [Text Word] OR GW 869682 [Text Word] OR sergliflozin etabonate [Text Word] OR sergliflozin [Text Word] OR EGT0001474 [Text Word] OR SHR3824 [Text Word] OR GSK-1614235 [Text Word] OR BI 44847 [Text Word]) |
| **Embase (8752)** | | |
|  | RCT strategy | ('randomized controlled trial'/exp OR 'randomized controlled trial') |
| AND | SGLT2i treatment | ('sodium glucose cotransporter 2'/exp OR 'sodium glucose cotransporter 2' OR 'sodium glucose cotransporter 2 inhibitor'/exp OR 'sodium glucose cotransporter 2 inhibitor' OR 'dapagliflozin'/exp OR 'dapagliflozin' OR 'farxiga' OR 'bms 512148' OR 'canagliflozin'/exp OR 'canagliflozin' OR 'invokana' OR 'jnj 28431754' OR 'empagliflozin'/exp OR 'empagliflozin' OR 'jardiance' OR 'bi 10773' OR 'ipragliflozin'/exp OR 'ipragliflozin' OR 'suglat' OR 'asp1941' OR 'luseogliflozin'/exp OR 'luseogliflozin' OR 'lusefi' OR 'ts-071' OR 'tofogliflozin'/exp OR 'tofogliflozin' OR 'apleway' OR 'deberza' OR 'ertugliflozin'/exp OR 'ertugliflozin' OR 'pf04971729' OR 'mk-8835' OR 'sotagliflozin'/exp OR 'sotagliflozin' OR 'lx4211' OR 'bexagliflozin' OR 'egt0001442' OR 'remogliflozinetabonate'/exp OR 'remogliflozin' OR 'gsk 189075' OR 'isis 388626' OR 'isis-sglt2rx' OR 'ta-7284' OR 'sergliflozinetabonate'/exp OR 'sergliflozin' OR 'gw 869682' OR 'egt0001474' OR 'shr3824' OR 'gsk-1614235' OR 'bi 44847'/exp OR 'bi 44847') |
| **Cochrane Central Register of Controlled Trials (7242)** | | |
|  | SGLT2i treatment | MeSH descriptor: [Sodium-Glucose Transporter 2] explode all trees OR  ALL TEXT= (“sodium-glucose transporter 2” OR “SGLT2” OR “SGLT-2” OR “Dapagliflozin or Farxiga or BMS-512148” OR “Canagliflozin or Invokana or JNJ-28431754” OR “Empagliflozin or Jardiance or BI 10773” OR “Ipragliflozin or Suglat or ASP1941” OR “Luseogliflozin or Lusefi or TS-071” OR “Tofogliflozin or Apleway or Deberza” OR “Ertugliflozin or PF04971729 or MK-8835” OR “Sotagliflozin or LX4211 or Zynquista” OR “Bexagliflozin or EGT0001442” OR “Remogliflozin or remogliflozin etabonate or GSK 189075” OR “ISIS 388626 or ISIS-SGLT2Rx” OR “TA-7284” OR “GW 869682 or sergliflozin etabonate or sergliflozin” OR “EGT0001474” OR “SHR3824” OR “GSK-1614235” OR “BI 44847”) |
| **Web of Science Core Collection (3471)** | | |
|  | RCT strategy | TS=("randomized controlled trial" OR "controlled clinical trial" OR "randomized" OR "clinical trial" OR "randomly" OR "trial" OR "placebo") |
| AND | SGLT2i treatment | TS=(“sodium-glucose transporter 2” OR “SGLT2” OR “SGLT-2” OR “Dapagliflozin or Farxiga or BMS-512148” OR “Canagliflozin or Invokana or JNJ-28431754” OR “Empagliflozin or Jardiance or BI 10773” OR “Ipragliflozin or Suglat or ASP1941” OR “Luseogliflozin or Lusefi or TS-071” OR “Tofogliflozin or Apleway or Deberza” OR “Ertugliflozin or PF04971729 or MK-8835” OR “Sotagliflozin or LX4211 or Zynquista” OR “Bexagliflozin or EGT0001442” OR “Remogliflozin or remogliflozin etabonate or GSK 189075” OR “ISIS 388626 or ISIS-SGLT2Rx” OR “TA-7284” OR “GW 869682 or sergliflozin etabonate or sergliflozin” OR “EGT0001474” OR “SHR3824” OR “GSK-1614235” OR “BI 44847”) |
