# Supplementary material for: Effect of sodium-glucose cotransporter-2 inhibitors on fracture risk in patients with type 1 diabetes receiving insulin-based therapy: a meta-analysis
Source: PeerJ. 2026 Apr 16;14:e21087. doi: 10.7717/peerj.21087 (PMC13092229; doi:10.7717/peerj.21087)
Supplement: Supplemental Information 2 [file peerj-14-21087-s002.doc]

**Supplemental table S2. Sensitivity analysis (Mantel-Haenszel method, fixed-effect model; and Leave-one-out sensitivity analysis)**

| **Sensitivity analysis: Mantel-Haenszel method, fixed-effect model** | | | | | | | |
| --- | --- | --- | --- | --- | --- | --- | --- |
| Outcome | Effect estimates [95% CI] | | | P-Value | I^2^ (%) | | |
| Bone fracture | OR: 1.00 [0.66, 1.53] | | | P=0.99 | 0 | | |
| HbA1c | MD: -0.38 [-0.42, -0.34] | | | P<0.001 | 62 | | |
| FPG | SMD: -0.27 [-0.35, -0.20] | | | P<0.001 | 0 | | |
| TIR | MD: 10.69 [9.62, 11.76] | | | P<0.001 | 34 | | |
| TID | SMD: -0.51 [-0.56, -0.46] | | | P<0.001 | 86 | | |
| Daily basal ID | SMD: -0.44 [-0.51, -0.37] | | | P<0.001 | 84 | | |
| Daily bolus ID | SMD: -0.24 [-0.31, -0.17] | | | P<0.001 | 56 | | |
| Bodyweight | SMD: -0.74 [-0.79, -0.68] | | | P<0.001 | 92 | | |
| SysBP | MD: -2.38 [-2.91, -1.85] | | | P<0.001 | 62 | | |
| DBP | MD: -1.36 [-1.79, -0.94] | | | P<0.001 | 0 | | |
| eGFR | SMD: -0.07 [-0.15, 0.01] | | | P=0.07 | 0 | | |
| ACR | MD: -11.74 [-25.79, 2.31] | | | P=0.10 | 18 | | |
| Diabetic eye disorders | OR: 0.51 [0.20, 1.28] | | | P=0.15 | 6 | | |
| DKA | OR: 4.08 [2.53, 6.58] | | | P<0.001 | 0 | | |
| MACE | OR: 1.06 [0.59, 1.91] | | | P=0.85 | 0 | | |
| Hypoglycemia | OR: 1.12 [0.96, 1.31] | | | P=0.14 | 9 | | |
| UTIs | OR: 1.00 [0.81, 1.23] | | | P=0.99 | 0 | | |
| GTIs | OR: 3.77 [2.91, 4.88] | | | P<0.001 | 0 | | |
| **Leave-one-out sensitivity analysis for Bone fracture** | | | | | | | |
| Study removed | | OR [95% CI] | P-Value | | | I^2^ (%) |  |
| B. W. Bode2021 | | 1.00 [0.64, 1.54] | P=0.99 | | | 0 |  |
| C.Baker2019 | | 0.99 [0.64, 1.53] | P=0.96 | | | 0 |  |
| C. Mathieu2020 | | 0.89 [0.56, 1.43] | P=0.64 | | | 0 |  |
| J. B. Buse2018 | | 1.10 [0.66, 1.84] | P=0.70 | | | 0 |  |
| J. Rosenstock(EASE-2)2018 | | 0.98 [0.63, 1.52] | P=0.91 | | | 0 |  |
| J. Rosenstock(EASE-3)2018 | | 0.97 [0.62, 1.50] | P=0.88 | | | 0 |  |
| K. Kaku2020 | | 0.98 [0.63, 1.51] | P=0.92 | | | 0 |  |
| P. Dandona2018 | | 0.88 [0.55, 1.41] | P=0.60 | | | 0 |  |
| S. K. Garg2017 | | 0.95 [0.60, 1.50] | P=0.82 | | | 0 |  |
| T. Danne2018 | | 1.09 [0.67, 1.77] | P=0.74 | | | 0 |  |
| **Leave-one-out sensitivity analysis for HbA1c** | | | | | | |  |
| Study removed | | MD [95% CI] | P-Value | | | I^2^ (%) |  |
| B. W. Bode2021 | | -0.37 [-0.44, -0.29] | P<0.001 | | | 66 |  |
| C.Baker2019 | | -0.36 [-0.43, -0.29] | P<0.001 | | | 66 |  |
| C. Mathieu2020 | | -0.38 [-0.45, -0.32] | P<0.001 | | | 54 |  |
| J. B. Buse2018 | | -0.37 [-0.45, -0.30] | P<0.001 | | | 60 |  |
| J. Rosenstock (EASE-2)2018 | | -0.34 [-0.40, -0.28] | P<0.001 | | | 47 |  |
| J. Rosenstock (EASE-3)2018 | | -0.36 [-0.43, -0.28] | P<0.001 | | | 66 |  |
| K. Kaku2020 | | -0.36 [-0.43, -0.29] | P<0.001 | | | 66 |  |
| P. Dandona2018 | | -0.36 [-0.44, -0.29] | P<0.001 | | | 66 |  |
| S. K. Garg2017 | | -0.35 [-0.42, -0.27] | P<0.001 | | | 58 |  |
| T. Danne2018 | | -0.37 [-0.44, -0.30] | P<0.001 | | | 61 |  |
| **Leave-one-out sensitivity analysis for FPG** | | | | | | |  |
| Study removed | | SMD [95% CI] | P-Value | | | I^2^ (%) |  |
| B. W. Bode2021 | | -0.27 [-0.35, -0.19] | P<0.001 | | | 4 |  |
| C.Baker2019 | | -0.27 [-0.35, -0.20] | P<0.001 | | | 4 |  |
| C. Mathieu2020 | | -0.27 [-0.36, -0.18] | P<0.001 | | | 4 |  |
| J. B. Buse2018 | | -0.28 [-0.37, -0.19] | P<0.001 | | | 4 |  |
| K. Kaku2020 | | -0.26 [-0.33, -0.18] | P<0.001 | | | 0 |  |
| P. Dandona2018 | | -0.26 [-0.35, -0.17] | P<0.001 | | | 0 |  |
| T. Danne2018 | | -0.31 [-0.39, -0.22] | P<0.001 | | | 0 |  |
| **Leave-one-out sensitivity analysis for TIR** | | | | | | |  |
| Study removed | | MD [95% CI] | P-Value | | | I^2^ (%) |  |
| B. W. Bode2021 | | 10.50 [8.89, 12.11] | P<0.001 | | | 43 |  |
| C. Mathieu2020 | | 10.48 [8.45, 12.52] | P<0.001 | | | 40 |  |
| J. B. Buse2018 | | 10.74 [9.34, 12.13] | P<0.001 | | | 26 |  |
| J. Rosenstock (EASE-2)2018 | | 9.64 [8.28, 11.00] | P<0.001 | | | 0 |  |
| P. Dandona2018 | | 10.48 [8.46, 12.49] | P<0.001 | | | 41 |  |
| T. Danne2018 | | 10.31 [8.62, 12.00] | P<0.001 | | | 47 |  |
| **Leave-one-out sensitivity analysis for TID** | | | | | | |  |
| Study removed | | SMD [95% CI] | P-Value | | | I^2^ (%) |  |
| B. W. Bode2021 | | -0.58 [-0.74, -0.42] | P<0.001 | | | 88 |  |
| C. Mathieu2020 | | -0.56 [-0.74, -0.38] | P<0.001 | | | 88 |  |
| J. B. Buse2018 | | -0.57 [-0.75, -0.40] | P<0.001 | | | 88 |  |
| J. Rosenstock (EASE-2)2018 | | -0.52 [-0.66, -0.37] | P<0.001 | | | 83 |  |
| J. Rosenstock (EASE-3)2018 | | -0.54 [-0.71, -0.37] | P<0.001 | | | 86 |  |
| K. Kaku2020 | | -0.50 [-0.63, -0.36] | P<0.001 | | | 82 |  |
| P. Dandona2018 | | -0.57 [-0.75, -0.40] | P<0.001 | | | 88 |  |
| S. K. Garg2017 | | -0.58 [-0.76, -0.41] | P<0.001 | | | 86 |  |
| T. Danne2018 | | -0.60 [-0.76, -0.44] | P<0.001 | | | 85 |  |
| **Leave-one-out sensitivity analysis for Daily basal ID** | | | | | | |  |
| Study removed | | SMD [95% CI] | P-Value | | | I^2^ (%) |  |
| B. W. Bode2021 | | -0.53 [-0.73, -0.33] | P<0.001 | | | 86 |  |
| C. Mathieu2020 | | -0.47 [-0.69, -0.26] | P<0.001 | | | 84 |  |
| J. B. Buse2018 | | -0.51 [-0.75, -0.27] | P<0.001 | | | 87 |  |
| K. Kaku2020 | | -0.41 [-0.54, -0.28] | P<0.001 | | | 67 |  |
| S. K. Garg2017 | | -0.54 [-0.78, -0.30] | P<0.001 | | | 85 |  |
| T. Danne2018 | | -0.55 [-0.77, -0.32] | P<0.001 | | | 85 |  |
| **Leave-one-out sensitivity analysis for Daily bolus ID** | | | | | | |  |
| Study removed | | SMD [95% CI] | P-Value | | | I^2^ (%) |  |
| B. W. Bode2021 | | -0.26 [-0.39, -0.14] | P<0.001 | | | 64 |  |
| C. Mathieu2020 | | -0.27 [-0.42, -0.13] | P<0.001 | | | 64 |  |
| J. B. Buse2018 | | -0.25 [-0.39, -0.11] | P<0.001 | | | 63 |  |
| K. Kaku2020 | | -0.22 [-0.29, -0.15] | P<0.001 | | | 0 |  |
| S. K. Garg2017 | | -0.27 [-0.43, -0.11] | P<0.001 | | | 64 |  |
| T. Danne2018 | | -0.29 [-0.41, -0.17] | P<0.001 | | | 50 |  |
| **Leave-one-out sensitivity analysis for Bodyweight** | | | | | | |  |
| Study removed | | SMD [95% CI] | P-Value | | | I^2^ (%) |  |
| B. W. Bode2021 | | -0.76 [-0.98, -0.54] | P<0.001 | | | 93 |  |
| C.Baker2019 | | -0.78 [-1.00, -0.56] | P<0.001 | | | 93 |  |
| C. Mathieu2020 | | -0.75 [-0.97, -0.53] | P<0.001 | | | 92 |  |
| J. B. Buse2018 | | -0.75 [-0.97, -0.53] | P<0.001 | | | 93 |  |
| J. Rosenstock (EASE-2)2018 | | -0.83 [-1.03, -0.64] | P<0.001 | | | 90 |  |
| J. Rosenstock (EASE-3)2018 | | -0.83 [-1.03, -0.63] | P<0.001 | | | 90 |  |
| K. Kaku2020 | | -0.73 [-0.94, -0.51] | P<0.001 | | | 93 |  |
| P. Dandona2018 | | -0.78 [-1.02, -0.55] | P<0.001 | | | 93 |  |
| S. K. Garg2017 | | -0.75 [-0.97, -0.53] | P<0.001 | | | 92 |  |
| T. Danne2018 | | -0.79 [-1.02, -0.56] | P<0.001 | | | 93 |  |
| **Leave-one-out sensitivity analysis for SysBP** | | | | | | |  |
| Study removed | | MD [95% CI] | P-Value | | | I^2^ (%) |  |
| C. Mathieu2020 | | -2.64 [-3.62, -1.65] | P<0.001 | | | 67 |  |
| J. B. Buse2018 | | -2.57 [-3.63, -1.50] | P<0.001 | | | 64 |  |
| J. Rosenstock (EASE-2)2018 | | -2.94 [-4.12, -1.76] | P<0.001 | | | 66 |  |
| J. Rosenstock (EASE-3)2018 | | -3.03 [-3.83, -2.23] | P<0.001 | | | 25 |  |
| P. Dandona2018 | | -2.64 [-3.72, -1.57] | P<0.001 | | | 67 |  |
| S. K. Garg2017 | | -2.37 [-3.29, -1.44] | P<0.001 | | | 47 |  |
| T. Danne2018 | | -2.71 [-3.84, -1.57] | P<0.001 | | | 68 |  |
| Leave-one-out sensitivity analysis for DBP | | | | | | |  |
| Study removed | | MD [95% CI] | P-Value | | | I^2^ (%) |  |
| J. B. Buse2018 | | -1.27 [-1.73, -0.81] | P<0.001 | | | 0 |  |
| J.Rosenstock (EASE-2)2018 | | -1.30 [-1.76, -0.85] | P<0.001 | | | 0 |  |
| J. Rosenstock (EASE-3)2018 | | -1.40 [-1.88, -0.92] | P<0.001 | | | 0 |  |
| P. Dandona2018 | | -1.40 [-1.84, -0.95] | P<0.001 | | | 0 |  |
| S. K. Garg2017 | | -1.43 [-1.89, -0.97] | P<0.001 | | | 0 |  |
| T. Danne2018 | | -1.39 [-1.89, -0.89] | P<0.001 | | | 0 |  |
| Leave-one-out sensitivity analysis for eGFR | | | | | | |  |
| Study removed | | SMD [95% CI] | P-Value | | | I^2^ (%) |  |
| B. W. Bode2021 | | -0.07 [-0.14, 0.01] | P=0.09 | | | 0 |  |
| C.Baker2019 | | -0.07 [-0.15, 0.01] | P=0.07 | | | 0 |  |
| J. B. Buse2018 | | -0.06 [-0.15, 0.03] | P=0.19 | | | 0 |  |
| S. K. Garg2017 | | -0.12 [-0.23, -0.01] | P=0.03 | | | 0 |  |
| T. Danne2018 | | -0.06 [-0.14, 0.03] | P=0.20 | | | 0 |  |
| **Leave-one-out sensitivity analysis for ACR** | | | | | | |  |
| Study removed | | MD [95% CI] | P-Value | | | I^2^ (%) |  |
| J. B. Buse2018 | | -18.77 [-35.37, -2.16] | P=0.03 | | | 0 |  |
| S. K. Garg2017 | | -7.24 [-30.46, 15.98] | P=0.54 | | | 47 |  |
| T. Danne2018 | | -7.11 [-32.62, 18.41] | P=0.59 | | | 47 |  |
| **Leave-one-out sensitivity analysis for Diabetic eye disorders** | | | | | | |  |
| Study removed | | OR [95% CI] | P-Value | | | I^2^ (%) |  |
| J.Rosenstock(EASE-2)2018 | | 0.53 [0.13, 2.17] | P=0.38 | | | 23 |  |
| J.Rosenstock(EASE-3)2018 | | 0.64 [0.20, 2.03] | P=0.45 | | | 0 |  |
| P. Dandona2018 | | 0.27 [0.08, 0.96] | P=0.04 | | | 0 |  |
| S. K. Garg2017 | | 0.47 [0.12, 1.87] | P=0.28 | | | 29 |  |
| T. Danne2018 | | 0.43 [0.09, 2.05] | P=0.29 | | | 30 |  |
| **Leave-one-out sensitivity analysis for DKA** | | | | | | |  |
| Study removed | | OR [95% CI] | P-Value | | | I^2^ (%) |  |
| C. Mathieu2020 | | 3.28 [1.99, 5.40] | P<0.001 | | | 0 |  |
| J. B. Buse2018 | | 3.28 [1.99, 5.41] | P<0.001 | | | 0 |  |
| J. Rosenstock (EASE-2)2018 | | 3.81 [2.21, 6.55] | P<0.001 | | | 0 |  |
| J. Rosenstock (EASE-3)2018 | | 3.80 [2.24, 6.44] | P<0.001 | | | 0 |  |
| K. Kaku2020 | | 3.61 [2.19, 5.93] | P<0.001 | | | 0 |  |
| P. Dandona2018 | | 4.07 [2.33, 7.10] | P<0.001 | | | 0 |  |
| S. K. Garg2017 | | 3.15 [1.83, 5.42] | P<0.001 | | | 0 |  |
| T. Danne2018 | | 3.36 [2.05, 5.50] | P<0.001 | | | 0 |  |
| **Leave-one-out sensitivity analysis for MACE** | | | | | | |  |
| Study removed | | OR [95% CI] | P-Value | | | I^2^ (%) |  |
| C. Mathieu2020 | | 1.11 [0.53, 2.34] | P=0.78 | | | 4 |  |
| J. B. Buse2018 | | 1.30 [0.59, 2.88] | P=0.51 | | | 0 |  |
| J.Rosenstock(EASE-2)2018 | | 0.94 [0.47, 1.88] | P=0.87 | | | 3 |  |
| J.Rosenstock(EASE-3)2018 | | 0.94 [0.49, 1.83] | P=0.86 | | | 1 |  |
| K. Kaku2020 | | 1.11 [0.57, 2.14] | P=0.76 | | | 0 |  |
| P. Dandona2018 | | 0.94 [0.45, 1.97] | P=0.88 | | | 5 |  |
| S. K. Garg2017 | | 0.92 [0.48, 1.78] | P=0.81 | | | 0 |  |
| T. Danne2018 | | 0.89 [0.45, 1.75] | P=0.73 | | | 0 |  |
| **Leave-one-out sensitivity analysis for Hypoglycemia** | | | | | | |  |
| Study removed | | OR [95% CI] | P-Value | | | I^2^ (%) |  |
| B. W. Bode2021 | | 1.14 [0.94, 1.38] | P=0.19 | | | 19 |  |
| C.Baker2019 | | 1.14 [0.95, 1.37] | P=0.16 | | | 14 |  |
| C. Mathieu2020 | | 1.18 [0.97, 1.42] | P=0.09 | | | 8 |  |
| J. B. Buse2018 | | 1.14 [0.96, 1.37] | P=0.14 | | | 13 |  |
| J.Rosenstock(EASE-2)2018 | | 1.19 [0.97, 1.45] | P=0.09 | | | 8 |  |
| J.Rosenstock(EASE-3)2018 | | 1.16 [0.92, 1.45] | P=0.21 | | | 18 |  |
| K. Kaku2020 | | 1.12 [0.95, 1.31] | P=0.17 | | | 0 |  |
| P. Dandona2018 | | 1.04 [0.87, 1.23] | P=0.70 | | | 0 |  |
| S. K. Garg2017 | | 1.12 [0.91, 1.37] | P=0.28 | | | 18 |  |
| T. Danne2018 | | 1.13 [0.93, 1.37] | P=0.22 | | | 18 |  |
| **Leave-one-out sensitivity analysis for UTIs** | | | | | | |  |
| Study removed | | OR [95% CI] | P-Value | | | I^2^ (%) |  |
| B. W. Bode2021 | | 0.99 [0.80, 1.22] | P=0.90 | | | 0 |  |
| C.Baker2019 | | 1.01 [0.82, 1.25] | P=0.92 | | | 0 |  |
| C. Mathieu2020 | | 0.98 [0.78, 1.23] | P=0.88 | | | 0 |  |
| J. B. Buse2018 | | 1.00 [0.79, 1.25] | P=0.98 | | | 0 |  |
| J.Rosenstock(EASE-2)2018 | | 1.01 [0.80, 1.28] | P=0.93 | | | 0 |  |
| J.Rosenstock(EASE-3)2018 | | 0.99 [0.79, 1.23] | P=0.90 | | | 0 |  |
| K. Kaku2020 | | 1.03 [0.83, 1.27] | P=0.81 | | | 0 |  |
| P. Dandona2018 | | 0.97 [0.77, 1.22] | P=0.80 | | | 0 |  |
| S. K. Garg2017 | | 1.01 [0.80, 1.27] | P=0.94 | | | 0 |  |
| T. Danne2018 | | 0.99 [0.79, 1.23] | P=0.90 | | | 0 |  |
| **Leave-one-out sensitivity analysis for GTIs** | | | | | | |  |
| Study removed | | OR [95% CI] | P-Value | | | I^2^ (%) |  |
| B. W. Bode2021 | | 3.68 [2.84, 4.78] | P<0.001 | | | 0 |  |
| C.Baker2019 | | 3.70 [2.85, 4.80] | P<0.001 | | | 0 |  |
| C. Mathieu2020 | | 3.79 [2.86, 5.01] | P<0.001 | | | 0 |  |
| J. B. Buse2018 | | 3.71 [2.81, 4.90] | P<0.001 | | | 0 |  |
| J.Rosenstock(EASE-2)2018 | | 3.78 [2.83, 5.06] | P<0.001 | | | 0 |  |
| J.Rosenstock(EASE-3)2018 | | 3.72 [2.84, 4.89] | P<0.001 | | | 0 |  |
| K. Kaku2020 | | 3.67 [2.83, 4.76] | P<0.001 | | | 0 |  |
| P. Dandona2018 | | 3.45 [2.62, 4.56] | P<0.001 | | | 0 |  |
| S. K. Garg2017 | | 3.83 [2.87, 5.11] | P<0.001 | | | 0 |  |
| T. Danne2018 | | 3.60 [2.74, 4.72] | P<0.001 | | | 0 |  |
