# Supplementary figures and images for: Effect of sodium-glucose cotransporter-2 inhibitors on fracture risk in patients with type 1 diabetes receiving insulin-based therapy: a meta-analysis
Source: PeerJ. 2026 Apr 16;14:e21087. doi: 10.7717/peerj.21087 (PMC13092229; doi:10.7717/peerj.21087)

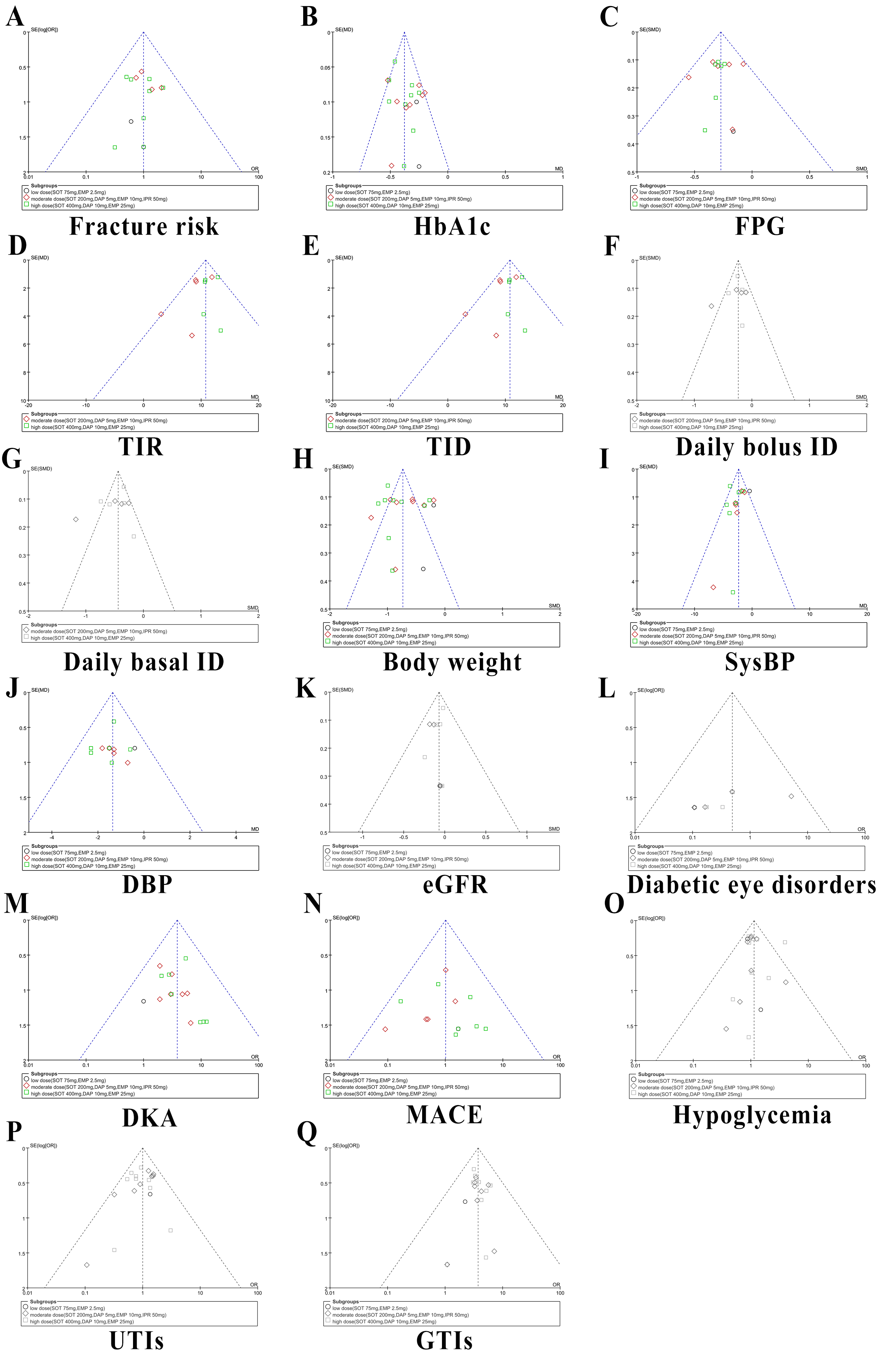

Supplement: Supplemental Information 3 — Publication bias for the outcomes. Outcomes: (A) fracture risk, (B) HbA1c, (C) Fasting plasma glucose ( FPG), (D) Time-in-range (70-180 mg/dl) (TIR), (E) Daily total insulin dose, (F) Daily bolus insulin dose, (G) Daily basal insulin dose, (H) Body weight changes, (I) Systolic b lood p ressure (SysBP), (J) Diastolic blood pressure (DBP), (K) Estimated glomerular filtration rate (eGFR), (L) Diabetic eye disorders, (M) Diabetic ketoacidosis (DKA), (N) Cardiovascular events (MACE), (O) Hypoglycemia, (P) Urinary tract infections (UTIs), (Q) Genital tract infections (GTIs). SE, standard error; MD, mean difference; SMD, standardized mean difference: OR, odds ratio. [file peerj-14-21087-s003.png]

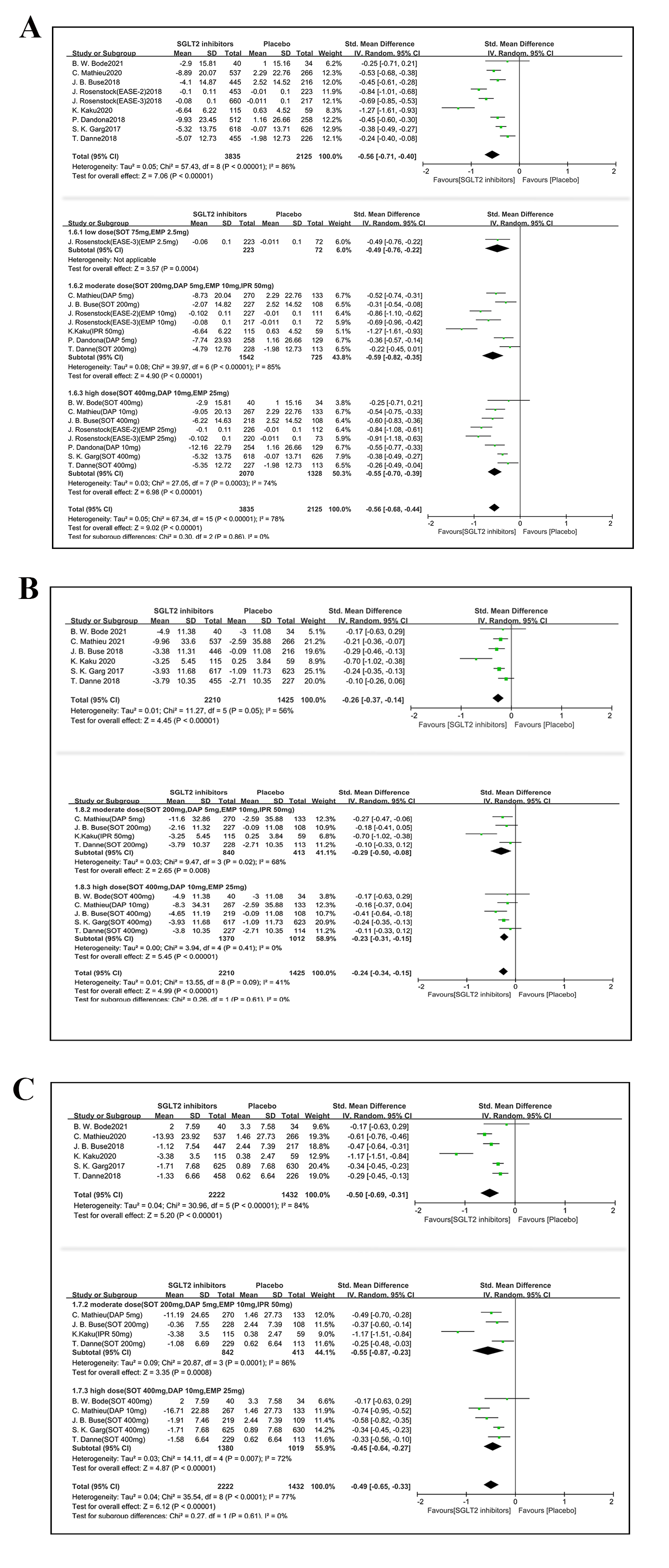

Supplement: Supplemental Information 4 — The effects of SGLT2i on glycemic efficacy outcomes with insulin therapy in T1DM. The primary analysis is above and the subgroup analysis is below for each plot. (A) Daily t otal insulin dose, (B) Daily bolus insulin dose, (C) Daily basal insulin dose, (D) hypoglycemia. SD, standard deviation; MD, mean average difference; SMD, standardized mean difference; M-H, Mantel-Haenszel; CI, 95% confidence intervals. [file peerj-14-21087-s004.png]

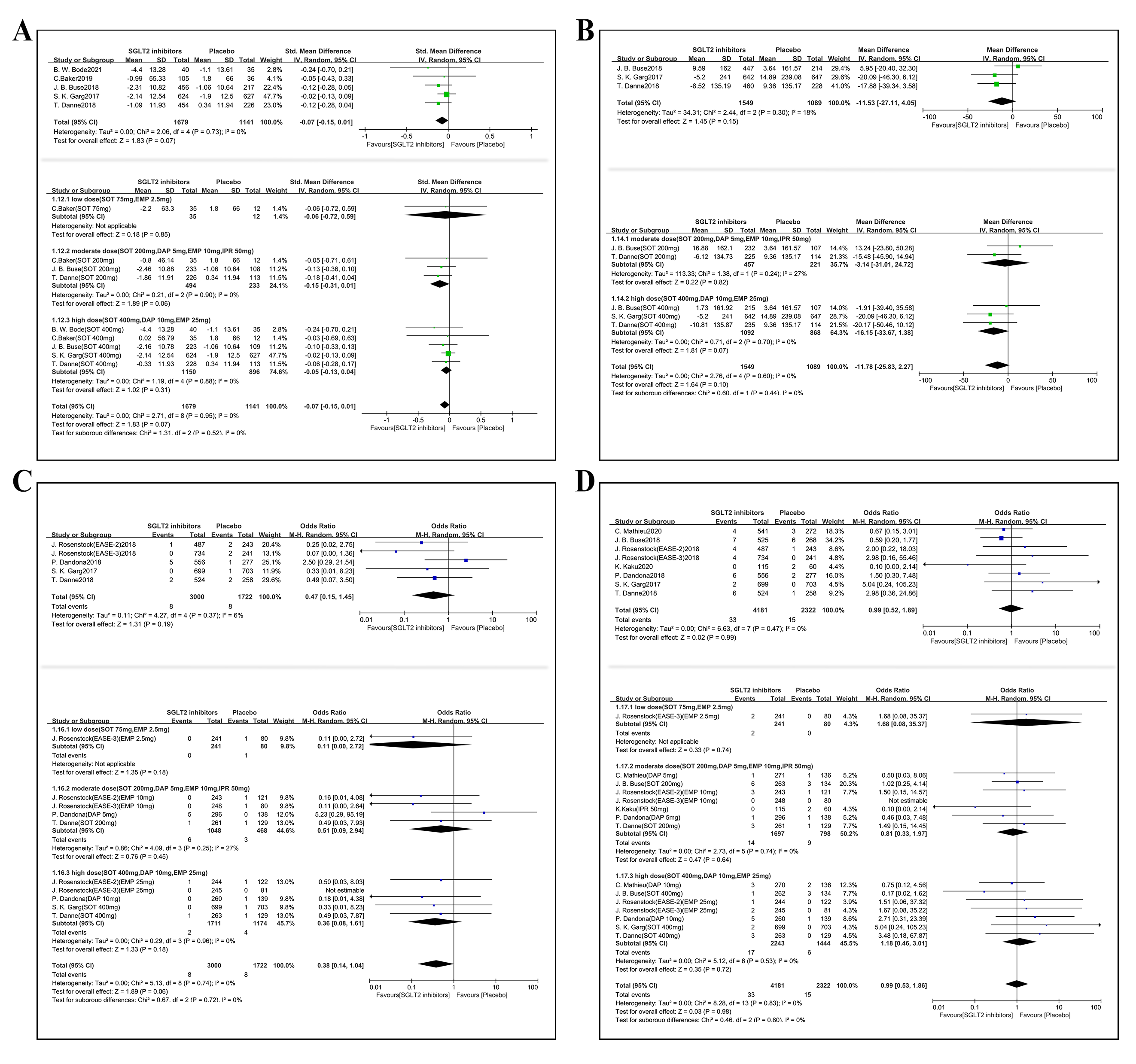

Supplement: Supplemental Information 5 — The effects of SGLT2i on four outcomes adding to insulin therapy in T1DM, compared with placebo group. The primary analysis is above and the subgroup analysis is below for each plot. (A) estimated glomerular filtration rate (eGFR), (B) urinary albumin to creatinine ratio (ACR), (C) diabetic eye disorders, (D) Cardiovascular events (MACE). SD, Standard deviation; MD, mean average difference; SMD, standardized mean difference; OR, Odds Ratio; M-H, Mantel-Haenszel; CI, 95% confidence intervals. [file peerj-14-21087-s005.png]
